# Effects of different types of written vaccination information on COVID-19 vaccine hesitancy in the UK (OCEANS-III): a single-blind, parallel-group, randomised controlled trial

**DOI:** 10.1016/S2468-2667(21)00096-7

**Published:** 2021-05-13

**Authors:** Daniel Freeman, Bao Sheng Loe, Ly-Mee Yu, Jason Freeman, Andrew Chadwick, Cristian Vaccari, Milensu Shanyinde, Victoria Harris, Felicity Waite, Laina Rosebrock, Ariane Petit, Samantha Vanderslott, Stephan Lewandowsky, Michael Larkin, Stefania Innocenti, Andrew J Pollard, Helen McShane, Sinéad Lambe

**Affiliations:** a Department of Psychiatry, University of Oxford, Oxford, UK; b Nuffield Department of Primary Care and Health Sciences, University of Oxford, Oxford, UK; c Oxford Vaccine Group, Department of Paediatrics, University of Oxford, Oxford, UK; d Smith School of Enterprise and the Environment, University of Oxford, Oxford, UK; e The Jenner Institute, Nuffield Department of Medicine, University of Oxford, Oxford, UK; f Oxford Health NHS Foundation Trust, Oxford, UK; g NIHR Oxford Health Biomedical Research Centre, Oxford, UK; h The Psychometrics Centre, University of Cambridge, Cambridge, UK; i Online Civic Culture Centre, Department of Communication and Media, Loughborough University, Loughborough, UK; j School of Psychological Science, University of Bristol, Bristol, UK; k Department of Psychology, Life and Health Sciences, Aston University, Birmingham, UK; l NIHR Oxford Biomedical Research Centre, Oxford, UK

## Abstract

**Background:**

The effectiveness of the COVID-19 vaccination programme depends on mass participation: the greater the number of people vaccinated, the less risk to the population. Concise, persuasive messaging is crucial, particularly given substantial levels of vaccine hesitancy in the UK. Our aim was to test which types of written information about COVID-19 vaccination, in addition to a statement of efficacy and safety, might increase vaccine acceptance.

**Methods:**

For this single-blind, parallel-group, randomised controlled trial, we aimed to recruit 15 000 adults in the UK, who were quota sampled to be representative. Participants were randomly assigned equally across ten information conditions stratified by level of vaccine acceptance (willing, doubtful, or strongly hesitant). The control information condition comprised the safety and effectiveness statement taken from the UK National Health Service website; the remaining conditions addressed collective benefit, personal benefit, seriousness of the pandemic, and safety concerns. After online provision of vaccination information, participants completed the Oxford COVID-19 Vaccine Hesitancy Scale (outcome measure; score range 7–35) and the Oxford Vaccine Confidence and Complacency Scale (mediation measure). The primary outcome was willingness to be vaccinated. Participants were analysed in the groups they were allocated. p values were adjusted for multiple comparisons. The study was registered with ISRCTN, ISRCTN37254291.

**Findings:**

From Jan 19 to Feb 5, 2021, 15 014 adults were recruited. Vaccine hesitancy had reduced from 26·9% the previous year to 16·9%, so recruitment was extended to Feb 18 to recruit 3841 additional vaccine-hesitant adults. 12 463 (66·1%) participants were classified as willing, 2932 (15·6%) as doubtful, and 3460 (18·4%) as strongly hesitant (ie, report that they will avoid being vaccinated for as long as possible or will never get vaccinated). Information conditions did not alter COVID-19 vaccine hesitancy in those willing or doubtful (adjusted p values >0·70). In those strongly hesitant, COVID-19 vaccine hesitancy was reduced, in comparison to the control condition, by personal benefit information (mean difference –1·49, 95% CI –2·16 to –0·82; adjusted p=0·0015), directly addressing safety concerns about speed of development (−0·91, –1·58 to –0·23; adjusted p=0·0261), and a combination of all information (−0·86, –1·53 to –0·18; adjusted p=0·0313). In those strongly hesitant, provision of personal benefit information reduced hesitancy to a greater extent than provision of information on the collective benefit of not personally getting ill (−0·97, 95% CI –1·64 to –0·30; adjusted p=0·0165) or the collective benefit of not transmitting the virus (−1·01, –1·68 to –0·35; adjusted p=0·0150). Ethnicity and gender were found to moderate information condition outcomes.

**Interpretation:**

In the approximately 10% of the population who are strongly hesitant about COVID-19 vaccines, provision of information on personal benefit reduces hesitancy to a greater extent than information on collective benefits. Where perception of risk from vaccines is most salient, decision making becomes centred on the personal. As such, messaging that stresses the counterbalancing personal benefits is likely to prove most effective. The messaging from this study could be used in public health communications. Going forwards, the study highlights the need for future health campaigns to engage with the public on the terrain that is most salient to them.

**Funding:**

National Institute for Health Research (NIHR) Oxford Biomedical Research Centre and NIHR Oxford Health Biomedical Research Centre.

## Introduction

In October, 2020, we did a survey of UK adults to estimate how many people would agree to be vaccinated for COVID-19, to discover whether there were parts of the population who were especially reluctant and, most importantly, to determine why people might not agree to take an approved vaccine.^1^ Our aim was to inform the provision of accurate vaccination information that will increase acceptance rates. 5114 adults, representative of the UK population for age, gender, ethnicity, income, and region, took part in the Oxford Coronavirus Explanations, Attitudes, and Narratives Survey II (OCEANS-II). We found that 72% of the population were willing to be vaccinated, 17% were doubtful, and 12% were strongly hesitant. Although vaccine hesitancy was associated with younger age, female gender, lower income, and ethnicity, it was spread over the whole of the population, and not confined to particular sociodemographic groups. What mattered most were the beliefs people held about a COVID-19 vaccine—specifically, the potential collective benefit, the likelihood of COVID-19 infection, the effectiveness of a vaccine, its side-effects, and the speed of vaccine development. Those who were hesitant about a COVID-19 vaccine tended to be people who were less aware of the public health benefits of vaccination, did not consider themselves at high risk of illness, doubted the efficacy of a vaccine, worried about potential side-effects, or feared that it had been developed too quickly. The factor most strongly associated with vaccine acceptance was the awareness of collective benefit. We used these insights to develop OCEANS-III, a randomised controlled trial to test the effect of different types of written information on willingness to be vaccinated.

**Research in context**

**Evidence before this study**

Following PRISMA guidelines, we searched MEDLINE, Embase, PsychInfo, and medRxiv from Jan 1, 2020, to Feb 24, 2021, using the search terms (COVID-19 OR Coronavirus OR SARS-CoV-2) AND (vaccine OR vaccination OR immunization) AND (hesitancy OR confidence OR uptake OR acceptance). We found 937 papers, of which four used a randomised controlled design to examine the effect of information provision on COVID-19 vaccine hesitancy. A US study done in August, 2020, with 1123 respondents found that providing information that a vaccine is safe and effective, compared with no information, increased vaccine acceptance. In a French study done in July, 2020, with a representative group of 1942 people, there was no overall effect on vaccine acceptance from the provision of information on the benefit of herd immunity. Within the group, however, those individuals who outright refused vaccination (28·8%) were not affected by information on the benefit of herd immunity, but for those who did not refuse vaccination outright (71·2%), information on the benefit of herd immunity increased acceptance slightly. In a German study with 1349 members of the general population done in November, 2020, vaccine acceptance was not influenced by information on the individual and prosocial benefits of herd immunity. In a study of 8000 people in the UK and the USA in September, 2020, provision of negative misinformation, compared with factual information, reduced acceptance.

**Added value of this study**

The overarching question addressed in our study was whether there is specific brief content about COVID-19 vaccination, above a statement of safety and effectiveness, that might reduce hesitancy or consolidate existing positive views in the general population. The study was done after the COVID-19 vaccination programme had started in the UK. It was a large study, testing multiple different conditions of interest, and included outcome tests, moderation, and mediation. The study shows that for individuals who are initially strongly hesitant (ie, will avoid being vaccinated for as long as possible or will never get vaccinated), the most effective message for reducing hesitancy highlighted the personal benefits of vaccination (eg, that vaccination can prevent serious illness or long-term COVID-19-related problems). Addressing personal benefit was better than addressing collective benefit. It is also helpful in this subgroup to directly address safety concerns about the speed of development of the vaccines. Mediation of effects by changes in underlying views about COVID-19 vaccines was not found. The various information conditions tested, including those that reduced hesitancy in the strongly hesitant, did not change hesitancy levels either in people who were already willing to be vaccinated or those who were doubtful (ie, those who would delay vaccination or who did not know whether they would agree to receive the vaccine). There might be differences in impact of messages by gender and ethnicity, with participants of Black ethnicity sometimes reacting oppositely (more negatively) to the information provision conditions in comparison with the statement of safety and efficacy.

**Implications of all the available evidence**

The overwhelming majority in the UK are willing to be vaccinated for COVID-19 and can appreciate the collective benefits. In the small proportion, approximately 10% at the time of the study, who are strongly COVID-19 vaccine hesitant, and less inclined to see the collective benefit of vaccination, it is likely to be better to highlight personal rather than collective benefit in information provision. This group also show greater concerns about the speed of development of the vaccines and are responsive to information designed to address these fears. The information emphasis that reduces hesitancy in this subgroup does not affect vaccination attitudes in the rest of the population. None of the messaging lowered hesitancy in those who were doubtful about vaccination; research is needed to identify messaging that is especially effective for this group. Research also needs to consider the moderation of messages by gender and ethnicity. This study provides clear text for information provision in UK vaccine programmes. It was not determined how the successful information worked or whether the study findings can be generalised to other countries.

There have been four previous online randomised controlled studies, conducted before the approval of COVID-19 vaccines, that assessed aspects of information provision and vaccine acceptance. Informing people that a COVID-19 vaccine is safe and effective increases acceptance compared with a no-information condition.^2^ Provision of information on the collective herd immunity benefit of vaccination has shown no overall population effect on vaccine acceptance.^3^, ^4^ However, in one study, those who refuse COVID-19 vaccines outright were unaffected by a statement of the collective benefit of vaccination, whereas the provision of such information was associated with slightly higher acceptance in the rest of the population.^4^ Moderation of the effects of different messages is highly plausible, especially by level of vaccine hesitancy. Negative misinformation, compared with factual information, has been found to reduce vaccine acceptance.^5^

In OCEANS-III, we assumed that a basic statement of efficacy and safety—reproduced from the UK National Health Service (NHS) website—should occur in all information provision. We then tested the effects of additional short sections of text that addressed collective benefits of vaccination (arising from not getting ill or not infecting others), personal benefits of vaccination, the seriousness of the virus, and the speed of development and testing of the vaccines. We tested pieces of information suitable for use online or in a brief single sheet of information as part of a vaccination programme. We also tested the effects of combining some of these messages. All messaging was designed to be accurate and to reduce hesitancy; there was no testing of information that we thought might hinder vaccine uptake. The primary outcome was willingness to be vaccinated. We aimed to test moderation of the effectiveness of messaging by hesitancy level and a number of sociodemographic factors. We also set out to test mediation of any effects by beliefs about COVID-19 vaccination.

## Methods

### Study design

This study was a single-blind, parallel-group, randomised controlled trial, with planned mediation and moderation tests. The intervention and data collection were carried out online. Participants completed an item for stratification by vaccine hesitancy level, provided sociodemographic information, read the vaccine information in their randomly allotted condition, and then completed measures of COVID-19 vaccine hesitancy and COVID-19 complacency and confidence beliefs. The study received ethical approval from the University of Oxford Central University Research Ethics Committee (reference R74001/RE001).

### Participants

We aimed to recruit 15 000 UK adults (≥18 years of age), who were quota sampled to be nationally representative for age, gender, region, education level, and ethnicity. The quotas were based on UK Office for National Statistics population estimates. The participants were recruited by the market research company Lucid. Lucid's platform serves as a centralised source for survey responses, working with more than 250 survey suppliers. Lucid operates a marketplace in which they advertise the survey to suppliers, who provide individual participants, with sampling by Lucid from this pool. The advantage of using multiple survey sources is substantially less reliance on any particular demographic or segment of the population. Respondents were sourced from a variety of places: advertisements and promotions across digital networks, internet search, word of mouth and membership referrals, social networks, online and mobile games, affiliate marketing, banner advertisements, offerwalls, TV and radio advertisements, and offline recruitment with mail campaigns. Invited respondents were not told the topic of the survey before they provided provisional agreement to complete it. The description of the purpose of the study provided to participants, after they had given provisional agreement, was as follows: “There are now approved vaccines for COVID-19 that will be rolled out in the UK over the coming months. We want to learn about people's views about vaccination for COVID-19. In particular, we want to find out how many people would or would not wish to be vaccinated and the reasons behind their decision.” On the front page of the study website, participants were provided with ethical committee-approved information describing the study and were asked to complete online tick-boxes to indicate informed consent.

Study recruitment initially took place from Jan 19 to Feb 5, 2021. Owing to a reduction in vaccine hesitancy compared with OCEANS-II, recruitment of vaccine-hesitant individuals was extended to Feb 18, 2021. The UK vaccination programme started on Dec 8, 2020, for which a priority list was used (eg, older adults, clinically extremely vulnerable individuals, front-line health and social care staff). By Jan 19, 2021, when study recruitment began, 4 609 740 first-dose vaccinations had been given in the UK and 460 625 second doses.^6^ Nearly 10% of the UK adult population had received a vaccination.

### Randomisation and masking

Participants were randomly assigned online equally to ten information conditions, stratified by three levels of vaccine hesitancy (willing, doubtful, or strongly hesitant). Randomisation was done using a computer-generated sequence. Participants were required to read their assigned information condition and, therefore, could not be masked to the fact they were given information. They were, however, unaware that other conditions existed. Participants completed the self-report outcome measures online, with study data collected by Lucid; therefore, the research team can be considered as masked in relation to outcome assessments. The research team had no contact with research participants.

### Procedures

After agreeing to take part, participants answered a single question to determine their level of vaccine hesitancy: “If the vaccine was available at my GP surgery I would: (1) get it as soon as possible, (2) get it when I have time, (3) delay getting it, (4) avoid getting it for as long as possible, (5) never get it, (6) don't know.” As in OCEANS-II, the level of vaccine hesitancy was defined as willing (answer 1 or 2), doubtful (answer 3 or 6), or strongly hesitant (answer 4 or 5). This question was found in OCEANS-II to have the highest load (0·95) on the COVID-19 vaccine hesitancy latent factor but for that precise reason was not included in the primary outcome measure.^1^ That is, the stratifying question was designed not to be repeated in the principal outcome variable.

Participants were then asked to read the text associated with their assigned information condition. Condition 1 was the control group, with the text comprising the safety and effectiveness statement taken from the NHS website (table 1).^7^ Three conditions addressed collective benefit only (conditions 2, 3, and 4), one condition addressed personal benefit only (condition 5), one condition combined collective and personal benefits (condition 9), one condition addressed the seriousness of the pandemic (condition 6), two conditions addressed safety concerns (conditions 7 and 8), and the final condition was a full combination addressing benefit, seriousness, and safety concerns (condition 10; table 1). The full text of each information condition is included in the appendix (pp 54–59). The added text for each specific condition (conditions 2, 3, 5, 6, 7, and 8) had a word count within 20% of the mean. We consulted individuals from the Oxford Vaccine Group's Public and Patient Involvement group for feedback on the written text.

**Table 1:** Information conditions

	**Description**
Condition 1 (control)	Safety and effectiveness statement taken from the NHS website: the text was “The coronavirus (COVID-19) vaccines are safe and effective, and give you the best protection against coronavirus. They have been approved by the independent Medicines and Healthcare products Regulatory Agency (MHRA)”.^7^ This control statement features at the end of all other conditions.
Condition 2 (collective benefit)	Adding the collective vaccination benefit of not personally getting ill
Condition 3 (collective benefit)	Adding the collective vaccination benefit of not transmitting the virus to others
Condition 4 (collective benefit)	Adding the collective vaccination benefits of not getting ill and not transmitting the virus (ie, combining conditions 2 and 3)
Condition 5 (personal benefit)	Adding the personal benefit of getting vaccinated
Condition 6 (seriousness)	Adding the seriousness of the pandemic (and that it is much more serious than seasonal influenza)
Condition 7 (safety, direct)	Directly addressing concerns about vaccine safety related to speed of development
Condition 8 (safety, indirect)	Indirectly addressing concerns about vaccine safety related to speed of development
Condition 9 (collective and personal benefit)	Adding the collective and personal benefits together (ie, combining conditions 4 and 5)
Condition 10 (full combination)	Adding the information on the collective and personal benefits, the seriousness of the virus, and the information that indirectly addresses concerns about the speed of development (ie, combining conditions 4, 5, 6, and 8)

The full text for each information condition is included in the appendix (pp 54–59). NHS=National Health Service.

After reading the text associated with their assigned information condition, participants completed a measure of COVID-19 vaccine hesitancy (outcome measure) and a measure of COVID-19 complacency and confidence beliefs (mediation measure). The outcome measure was the seven-item Oxford COVID-19 Vaccine Hesitancy Scale,^1^ in which item-specific response options,^6^ coded from 1 to 5, are used. A “don't know” option is also provided, which is excluded from scoring. Scores can range between 7 and 35, with higher scores indicating higher COVID-19 vaccine hesitancy. The scale was developed in consultation with members of the general public, including representatives of ethnic minority groups. 14 items were initially developed and completed by the OCEANS-II representative sample of 5114 UK adults. Exploratory and confirmatory factor analysis (CFA) was conducted to derive the seven-item scale. The seven-item scale produced an excellent CFA model fit on the OCEANS-II sample (full-information maximum likelihood χ^2^(14, n=2548) = 93·370, p<0·0001; root mean square error of approximation 0·047; standardised root mean residual 0·01; comparative fit index 0·993; Tucker-Lewis index 0·989). Convergent validity was shown against the Vaccine Hesitancy Scale,^8^ Vaccination Knowledge Scale,^9^ and Vaccine Conspiracy Beliefs Scale.^10^ The Cronbach's α for the Oxford COVID-19 Vaccine Hesitancy Scale in the current study was 0·98 (n=16 445), indicating high internal consistency.

The mediation measure was the 14-item Oxford Vaccine Confidence and Complacency Scale,^1^ which has four subscale scores (collective importance, efficacy, side-effects, and speed of development). Item-specific response options,^11^ coded from 1 to 5, are used. A “don't know” option is also provided, which is excluded from scoring. Scores range from 14 to 60, with higher scores indicating more negative attitudes towards COVID-19 vaccination. The Cronbach's α in the current study was 0·88 (n=14 661) for collective importance, 0·85 (n=15 407) for speed of development, 0·71 (n=14 105) for efficacy, and 0·78 (n=15 080) for side-effects. The Cronbach's α for all the scale items was 0·94 (n=10 584), indicating high internal consistency.

Participants provided sociodemographic information including age, gender, ethnicity, region of residence, level of education, employment status, income, housing situation, marital status, religious beliefs, and political orientation. Participants completed all steps of the trial in the same session.

### Outcomes

Our primary outcome was willingness to be vaccinated, as measured by the Oxford COVID-19 Vaccine Hesitancy Scale. We considered two specific primary outcome questions: (1) whether adding information about the collective benefit of vaccination from not getting ill, the collective benefit of vaccination from not spreading the virus, the personal benefit of getting vaccinated, the seriousness of SARS-CoV-2, or why speed of development is not a problem (directly and indirectly) lead to lower levels of COVID-19 vaccine hesitancy compared with a simple statement that vaccination is efficacious and safe; and (2) whether combining information about collective and personal benefits, or about collective and personal benefits, the seriousness of the virus, and indirectly why the speed of development is not a problem, lead to lower levels of COVID-19 vaccine hesitancy than a simple statement that vaccination is efficacious and safe.

As part of our primary analysis, we also investigated whether the effect of information provision on COVID-19 vaccine hesitancy was moderated by the three levels of hesitancy groupings (ie, willing to be vaccinated, doubtful, strongly hesitant). We were most interested in the effects on those in the general population who were doubtful (approximately 17% in OCEANS-II) or strongly hesitant (approximately 12%) about a COVID-19 vaccination.

There were four specific secondary outcome questions: (1) whether emphasising collective benefit is better (ie, leads to lower hesitancy) than emphasising personal benefit; (2) whether it is better to address why speed of development is not a problem either directly or indirectly; (3) whether combining information about personal and collective benefits is better than emphasising either personal or collective benefits alone; and (4) whether combining information about collective and personal benefits with the seriousness of the virus and indirectly why the speed of development is not a problem is better than just combining information about collective and personal benefits.

As part of our secondary analysis, we also investigated whether the effect of information provision on COVID-19 vaccine hesitancy was moderated by age, gender, ethnicity, income, region, or level of COVID-19 health risk.

Finally, we considered the following mediation question: if a significant relationship exists between randomised information conditions and vaccine hesitancy, can that relationship be explained by COVID-19 vaccine views?

Adverse events were considered unlikely to occur (since we provided information that was accurate and aimed to encourage vaccine uptake). On the study's opening webpage, participants were informed that if they had a concern about any aspect of the study that they should contact the lead author or the Chair of the Medical Sciences Interdivisional Research Ethics Committee at the University of Oxford. Contact details for both individuals were provided.

### Statistical analysis

This study was powered to detect a change in hesitancy level for each of the three stratifying levels of hesitancy level (willing, doubtful, and strongly hesitant). From OCEANS-II, we estimated that 71·7% of the general population are willing to be vaccinated, with a mean score on the Oxford COVID-19 Hesitancy Scale of 10·8 (SD 3·5); 16·6% are doubtful, with a mean score of 24·2 (5·8); and 11·7% are strongly hesitant, with a mean score of 30·6 (3·5). A sample size of 96 would be able to detect a three-point change in the doubtful group, and a sample size of 254 a three-point change in the strongly hesitant group, at 90% power and a type 1 error at 0·5% (two-sided). For the willing groups, a sample size of 822 would be able to detect a one-point change. This gives a total sample size of 1172 required for each condition. We intended to recruit 15 000 participants to enable adjustment for multiple comparisons in the analysis.

The primary outcome, the Oxford COVID-19 Vaccine Hesitancy score, was analysed using a linear regression model. The linear regression model included the Oxford COVID-19 Vaccine Hesitancy Scale score as the response variable with the randomised group (conditions 2, 3, 4, 5, 6, 7, 8, 9, and 10 *vs* condition 1 [control]). An interaction between baseline vaccine hesitancy (willing, doubtful, or strongly hesitant) and condition was fitted in the regression model to determine the effect of conditions in each hesitancy category. Predefined subgroup analyses were done on the Oxford COVID-19 Vaccine Hesitancy Scale to ascertain if effectiveness of specific information conditions varied by individual characteristics (age, gender, ethnicity, income, region, and level of COVID-19 health risk). Subgroup analyses were conducted by inclusion of an interaction term of baseline subgroup by randomised group in separate regression models. Each of the regression models included the interaction term, adjusting for age, gender, ethnicity, income, region, level of COVID-19 health risk, and vaccine hesitancy level at baseline. p values and 95% CIs for the model coefficients were obtained by bootstrapping. To ensure a familywise error rate at 5%, p values were adjusted using the false discovery rate method.^12^

If an individual responded “don't know” to an item in the primary outcome measure, then they were excluded from the main analysis. For a sensitivity analysis concerning missing data, missing data measuring vaccine hesitancy at the item level were assumed to be missing at random and thus multiple imputation was used.^13^ Predictive mean matching was used as the imputation algorithm, which is appropriate for numerical data. The conditional predictive distribution of the item-level responses required to be imputed were adjusted to account for the information from age, gender, and level of COVID-19 health risk, all of which were known to be significant predictors of vaccine hesitancy as described in OCEANS-II.^1^ Only complete cases were used for age, gender, and level of COVID-19 health risk. Multiple imputation was imputed 50 times at the item level, and the responses from each imputed dataset were summed to create total hesitancy scores. The regression model was estimated 50 times and the predictions were averaged on the basis of Rubin's rules^14^ to calculate the estimated marginal means.

Mediation analyses were carried out using structural equation modelling. The factor scores of the Oxford COVID-19 Vaccine Hesitancy Scale were the response variable with randomised group as the exposure and a higher-order latent variable (beliefs) derived from the four subscales of the Oxford Vaccine Confidence and Complacency Scale as the mediator variable. Age, gender, ethnicity, income, region, and level of COVID-19 health risk were included in the model as controls. We employed the Monte Carlo method for a formal inferential test of the effects in the mediation model.^15^

A statistical analysis plan was finalised before the unblinding of data for analysis (appendix pp 2–9). There were no interim analyses. In a post-hoc analysis, we excluded participants who had been vaccinated from the outcome analyses, to assess whether their inclusion biased the results. For all analyses, participants were analysed in the groups they were allocated. Results were conducted in R (version 4.0.0) and then validated by a second statistician (L-MY) in Stata SE (version 16.1). This trial was prospectively registered with ISRCTN, ISRCTN37254291.

### Role of the funding source

Funding was provided by the National Institute for Health Research (NIHR) Oxford Biomedical Research Centre and the NIHR Oxford Health Biomedical Research Centre. The two funders had no role in study design, data collection, data analysis, data interpretation, or writing of the report.

## Results

From Jan 19 to Feb 5, 2021, 15 014 adults were recruited as planned (figure). Vaccine hesitancy was notably lower than found in the OCEANS-II survey conducted in October, 2020 (table 2). The largest reduction was in rates of those who were doubtful, although there had also clearly been a fall in the numbers of those who were strongly hesitant. Therefore, without a break and before analysis of the outcome results, we continued recruitment of vaccine-hesitant individuals (ie, doubtful or strongly hesitant). Recruitment ended on Feb 18, 2021, which provided an additional 3841 participants. A summary of the sociodemographic characteristics of the participants is given in table 3, with a breakdown by randomisation conditions provided in the appendix (pp 11–13). The three stratification hesitancy groups differed significantly, as would be expected, in beliefs about the collective benefit, personal risk and efficacy, speed of development concerns, and side-effect concerns. With increasing hesitancy level (willing to doubtful to strongly hesitant) there are increasingly negative views on all subscales of the Oxford Vaccine Confidence and Complacency Scale (appendix p 53). 1795 participants from the representative sample reported being vaccinated for COVID-19 and their hesitancy scores were significantly lower than the rest of the sample (p<0·00001). All 18 855 participants completed the primary outcome and 2400 participants who did not complete all items of the Oxford COVID-19 Vaccine Hesitancy Scale were excluded, meaning 16 455 were included in the primary analysis (figure).

**Figure:**
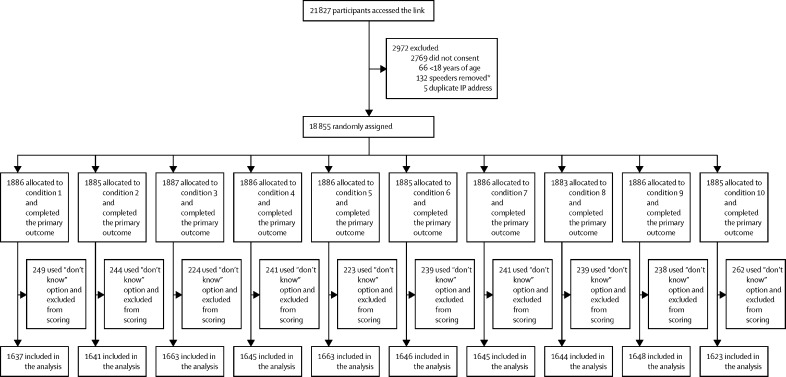
Trial profile IP=Internet Protocol. *Speeders defined as completion time of a third of the median length of interview, or below what is standard best practice.

**Table 2:** Vaccine hesitancy levels in the OCEANS-III and OCEANS-II cohorts

	**OCEANS-III cohort (n=18 855)**		**OCEANS-II cohort (n=5114)**
	Representative cohort (n=15 014)	Additional vaccine-hesitant group (n=3841)	
**If the vaccine was available at my GP surgery I would:**			
1. Get it as soon as possible	11 012 (73·3%)	0	2548 (49·8%)
2. Get it when I have time	1451 (9·7%)	0	1192 (23·3%)
3. Delay getting it	672 (4·5%)	976 (25·4%)	425 (8·3%)
4. Avoid getting it for as long as possible	771 (5·1%)	1164 (30·3%)	348 (6·8%)
5. Never get it	632 (4·2%)	893 (23·2%)	342 (6·7%)
6. Don't know	476 (3·2%)	808 (21·0%)	259 (5·1%)
**Stratification**			
Willing	12 463 (83·0%)	0	3740 (73·1%)
Doubtful	1148 (7·6%)	1784 (46·4%)	684 (13·4%)
Strongly hesitant	1403 (9·3%)	2057 (53·6%)	690 (13·5%)

The representative cohort was recruited from Jan 19 to Feb 5, 2021, with recruitment extended to Feb 18 for the additional vaccine-hesitant group. Participants in all cohorts are stratified according to their answers to the question displayed. GP=general practitioner.

**Table 3:** Baseline characteristics

			**Data (n=18 855)**
Age, years			43·2 (18·1)
Age group, years			
	18–24		3714 (19·7%)
	25–34		3611 (19·2%)
	35–44		3036 (16·1%)
	45–54		2835 (15·0%)
	55–64		2308 (12·2%)
	65–99		3351 (17·8%)
Gender			
	Female		10 512 (55·8%)
	Male		8155 (43·3%)
	Non-binary		86 (0·5%)
	Prefer not to say		102 (0·5%)
Ethnicity			
	White		
		English, Welsh, Scottish, Northern Irish, or British	14 053 (74·5%)
		Irish	229 (1·2%)
		Gypsy or Irish Traveller	83 (0·4%)
		Any other White background	915 (4·9%)
	Mixed or multiple ethnic groups		
		White and Black Caribbean	227 (1·2%)
		White and Black African	105 (0·6%)
		White and Asian	171 (0·9%)
		Any other mixed or multiple ethnic background	209 (1·1%)
	Asian or Asian British		
		Indian	435 (2·3%)
		Pakistani	378 (2·0%)
		Bangladeshi	218 (1·2%)
		Chinese	152 (0·8%)
		Any other Asian background	189 (1·0%)
	Black, African, Caribbean, or Black British		
		African	641 (3·4%)
		Caribbean	296 (1·6%)
		Any other Black, African, or Caribbean background	103 (0·5%)
	Other ethnic group		
		Arab	123 (0·7%)
		Any other ethnic group	97 (0·5%)
		Prefer not to say	231 (1·2%)
Marital status			
	Single		6684 (35·4%)
	Married or civil partnership		8145 (43·2%)
	Cohabiting		2290 (12·1%)
	Separated		763 (4·0%)
	Widowed		635 (3·4%)
	Prefer not to say		338 (1·8%)
Highest level of education			
	No qualifications		1252 (6·6%)
	GCSEs grades A*–C (or equivalent)		5256 (27·9%)
	AS levels (or equivalent)		795 (4·2%)
	A levels (or equivalent)		5118 (27·1%)
	Certificate of higher education (eg, BA, BSc, or equivalent)		4810 (25·5%)
	Postgraduate qualifications (eg, MA, MSc, PhD, DPhil)		1624 (8·6%)
Total household income			
	<£15 000		2973 (15·8%)
	£15 000–19 999		1867 (9·9%)
	£20 000–29 999		3325 (17·6%)
	£30 000–39 999		2682 (14·2%)
	£40 000–49 999		1934 (10·3%)
	£50 000–59 999		1367 (7·3%)
	£60 000–69 999		910 (4·8%)
	£70 000–99 999		1191 (6·3%)
	£100 000–149 999		610 (3·2%)
	£150 000 and above		301 (1·6%)
	Prefer not to say		1695 (9·0%)
Housing situation			
	Rented from council		3279 (17·4%)
	Rented from private landlord		4131 (21·9%)
	Homeowner		9938 (52·7%)
	Other		1507 (8·0%)
Region			
	North East		820 (4·3%)
	North West		2023 (10·7%)
	Yorkshire and the Humber		1488 (7·9%)
	East Midlands		1385 (7·3%)
	West Midlands		1796 (9·5%)
	East		1381 (7·3%)
	London		2936 (15·6%)
	South East		2605 (13·8%)
	South West		1509 (8·0%)
	Wales		892 (4·7%)
	Scotland		1528 (8·1%)
	Northern Ireland		492 (2·6%)
Pre-COVID-19 pandemic employment status			
	Unemployed		1693 (9·0%)
	Employed full time		7730 (41·0%)
	Employed part time		2732 (14·5%)
	Self-employed		1214 (6·4%)
	Retired		3156 (16·7%)
	Student		1335 (7·1%)
	Homemaker		995 (5·3%)
Employment change due to COVID-19 pandemic			
	None		10 466 (55·5%)
	None, but working from a different location (eg, working from home)		2589 (13·7%)
	Working hours have reduced		2032 (10·8%)
	Working hours have increased		609 (3·2%)
	Furlough		1444 (7·7%)
	Newly unemployed		1103 (5·8%)
	Newly employed (full time)		352 (1·9%)
	Newly employed (part time)		262 (1·4%)
Had COVID-19			
	Yes, a positive test		1273 (6·8%)
	No, a negative test		5972 (31·7%)
	Might have had it but not been tested		2173 (11·5%)
	Not had it but not been tested		9125 (48·4%)
	Other		312 (1·7%)
Risk for severe COVID-19 course			
	Low risk		11 121 (59·0%)
	Moderate risk		5204 (27·6%)
	Very high risk		1468 (7·8%)
	Do not know		1062 (5·6%)
Vaccinated for COVID-19			
	No		16 604 (88·1%)
	Yes, because in vulnerable group		886 (4·7%)
	Yes, because in key-worker group		883 (4·7%)
	Yes, for another reason		301 (1·6%)
	Do not know		181 (1·0%)

Data are n (%) or mean (SD).

We present the primary outcome results by stratification groups, since there was no indication of any main effect of condition (all adjusted p values >0·8; appendix pp 15–16) but there was moderation by vaccine hesitancy level (appendix p 19). The vaccine hesitancy estimated scores in each randomisation condition for each level of vaccine hesitancy are presented in table 4. Significant differences in vaccine hesitancy by information condition were only present for the strongly hesitant group, for whom condition 5 (personal benefits), condition 7 (directly addressing concerns about vaccine safety related to speed of development), and condition 10 (full combination) reduced vaccine hesitancy compared with the control condition (table 5). Condition 5 (personal benefits) led to the greatest reduction in hesitancy. There was no evidence that the differences by information condition were explained by changes in COVID-19 vaccine views (appendix pp 34–35).

**Table 4:** Estimated vaccine hesitancy marginal means in each information condition across the vaccine hesitancy groups

	**Number of participants**	**Estimated marginal mean (95% CI)**	**SE**
**Strongly hesitant**			
Condition 1 (control)	268	28·53 (28·05–29·01)	0·24
Condition 2 (collective benefit)	269	28·01 (27·54–28·49)	0·24
Condition 3 (collective benefit)	271	28·06 (27·58–28·53)	0·24
Condition 4 (collective benefit)	279	27·85 (27·38–28·32)	0·24
Condition 5 (personal benefit)	270	27·04 (26·57–27·51)	0·24
Condition 6 (seriousness)	263	28·29 (27·81–28·76)	0·25
Condition 7 (safety, direct)	261	27·62 (27·14–28·11)	0·25
Condition 8 (safety, indirect)	257	27·79 (27·30–28·28)	0·25
Condition 9 (collective and personal benefit)	284	28·17 (27·70–28·63)	0·24
Condition 10 (full combination)	264	27·67 (27·19–28·15)	0·24
**Doubtful**			
Condition 1 (control)	160	20·49 (19·87–21·10)	0·31
Condition 2 (collective benefit)	157	20·71 (20·09–21·33)	0·32
Condition 3 (collective benefit)	181	20·51 (19·93–21·09)	0·30
Condition 4 (collective benefit)	167	20·13 (19·53–20·73)	0·31
Condition 5 (personal benefit)	169	20·79 (20·19–21·39)	0·31
Condition 6 (seriousness)	170	20·08 (19·49–20·68)	0·30
Condition 7 (safety, direct)	170	20·60 (20·00–21·20)	0·30
Condition 8 (safety, indirect)	171	20·88 (20·29–21·48)	0·30
Condition 9 (collective and personal benefit)	163	19·87 (19·26–20·48)	0·31
Condition 10 (full combination)	143	20·80 (20·15–21·45)	0·33
**Willing**			
Condition 1 (control)	1209	9·39 (9·17–9·62)	0·11
Condition 2 (collective benefit)	1215	9·39 (9·16–9·61)	0·11
Condition 3 (collective benefit)	1211	9·37 (9·14–9·59)	0·11
Condition 4 (collective benefit)	1199	9·21 (8·98–9·43)	0·12
Condition 5 (personal benefit)	1224	9·24 (9·02–9·47)	0·11
Condition 6 (seriousness)	1213	9·42 (9·20–9·64)	0·11
Condition 7 (safety, direct)	1214	9·37 (9·15–9·59)	0·11
Condition 8 (safety, indirect)	1216	9·30 (9·08–9·53)	0·11
Condition 9 (collective and personal benefit)	1201	9·46 (9·23–9·68)	0·12
Condition 10 (full combination)	1216	9·18 (8·95–9·40)	0·11

Information conditions are outlined in table 1. Scores can range between 7 and 35, with higher scores indicating higher COVID-19 vaccine hesitancy.

**Table 5:** Mean difference between vaccine hesitancy marginal means for each randomised information conditions compared with control

	**Estimated mean difference in hesitancy (95% CI)**	**SE**	**Adjusted p value**
**Strongly hesitant**			
Condition 2 (collective benefit)	−0·51 (−1·19 to 0·16)	0·34	0·2171
Condition 3 (collective benefit)	−0·47 (−1·14 to 0·20)	0·34	0·2254
Condition 4 (collective benefit)	−0·68 (−1·35 to −0·01)	0·34	0·0846
Condition 5 (personal benefit)	−1·49 (−2·16 to −0·82)	0·34	0·0015
Condition 6 (seriousness)	−0·24 (−0·92 to 0·43)	0·35	0·5117
Condition 7 (safety, direct)	−0·91 (−1·58 to −0·23)	0·35	0·0261
Condition 8 (safety, indirect)	−0·74 (−1·42 to −0·06)	0·35	0·0703
Condition 9 (collective and personal benefit)	−0·36 (−1·03 to 0·30)	0·34	0·3514
Condition 10 (full combination)	−0·86 (−1·53 to −0·18)	0·34	0·0313
**Doubtful**			
Condition 2 (collective benefit)	0·23 (−0·65 to 1·10)	0·45	0·7656
Condition 3 (collective benefit)	0·02 (−0·82 to 0·87)	0·43	0·9615
Condition 4 (collective benefit)	−0·36 (−1·22 to 0·51)	0·44	0·7515
Condition 5 (personal benefit)	0·31 (−0·55 to 1·16)	0·44	0·7515
Condition 6 (seriousness)	−0·41 (−1·26 to 0·45)	0·44	0·7515
Condition 7 (safety, direct)	0·11 (−0·74 to 0·97)	0·44	0·9178
Condition 8 (safety, indirect)	0·40 (−0·46 to 1·25)	0·44	0·7515
Condition 9 (collective and personal benefit)	−0·62 (−1·48 to 0·25)	0·44	0·7515
Condition 10 (full combination)	0·32 (−0·58 to 1·21)	0·46	0·7515
**Willing**			
Condition 2 (collective benefit)	−0·01 (−0·32 to 0·31)	0·16	0·9741
Condition 3 (collective benefit)	−0·02 (−0·34 to 0·29)	0·16	0·9497
Condition 4 (collective benefit)	−0·19 (−0·50 to 0·13)	0·16	0·7443
Condition 5 (personal benefit)	−0·15 (−0·46 to 0·17)	0·16	0·7924
Condition 6 (seriousness)	0·03 (−0·29 to 0·34)	0·16	0·9497
Condition 7 (safety, direct)	−0·02 (−0·34 to 0·29)	0·16	0·9497
Condition 8 (safety, indirect)	−0·09 (−0·41 to 0·23)	0·16	0·9477
Condition 9 (collective and personal benefit)	0·06 (−0·25 to 0·38)	0·16	0·9477
Condition 10 (full combination)	−0·22 (−0·53 to 0·10)	0·16	0·7009

Estimated differences are between listed information conditions and the control group (ie, minus condition 1). Information conditions are outlined in table 1. Scores can range between 7 and 35, with higher scores indicating higher COVID-19 vaccine hesitancy.

In planned comparisons between conditions (other than control), we found that in the strongly hesitant group, personal benefit information was more effective than information on the collective benefit of not personally getting ill or the collective benefit of not transmitting the virus, and it was worse to combine personal and collective benefits than just to provide personal benefit information alone (table 6). These differences were not explained by changes in COVID-19 vaccine views (appendix pp 35–36). Addressing speed of development directly or indirectly did not differ in effect on vaccine hesitancy in any of the groups (table 6). There was no difference for any of the groups in effectiveness of combining all the available information (condition 10) compared with combining only information about collective and personal benefits (table 6).

**Table 6:** Mean difference between vaccine hesitancy marginal means across randomised information conditions

	**Estimated mean difference (95% CI)**	**SE**	**Adjusted p value**
**Personal benefit (condition 5) *vs* collective benefit of not getting ill (condition 2)**			
Strongly hesitant	−0·97 (−1·64 to −0·30)	0·34	0·0165
Doubtful	0·08 (−0·78 to 0·94)	0·44	0·9178
Willing	−0·14 (−0·46 to 0·17)	0·16	0·7924
**Personal benefit (condition 5) *vs* collective benefit of not transmitting (condition 3)**			
Strongly hesitant	−1·01 (−1·68 to −0·35)	0·34	0·0150
Doubtful	0·28 (−0·55 to 1·12)	0·43	0·7515
Willing	−0·12 (−0·44 to 0·19)	0·16	0·8211
**Addressing speed of development directly (condition 7) *vs* indirectly (condition 8)**			
Strongly hesitant	−0·17 (−0·52 to 0·85)	0·35	0·6355
Doubtful	0·28 (−0·56 to 1·13)	0·43	0·7515
Willing	−0·07 (−0·38 to 0·25)	0·16	0·9477
**Combining personal and collective benefits (condition 9) *vs* collective benefits alone (condition 4)**			
Strongly hesitant	0·32 (−0·34 to 0·97)	0·34	0·3980
Doubtful	−0·26 (−1·12 to 0·60)	0·44	0·7515
Willing	0·25 (−0·07 to 0·57)	0·16	0·7009
**Combining personal and collective benefits (condition 9) *vs* personal benefits alone (condition 5)**			
Strongly hesitant	1·12 (0·46 to 1·79)	0·34	0·0068
Doubtful	−0·92 (−1·78 to −0·07)	0·44	0·3015
Willing	0·21 (−0·10 to 0·53)	0·16	0·7009
**Combining collective and personal benefits, seriousness of the virus, and indirectly addressing safety concerns (condition 10) *vs* combining collective and personal benefits (condition 9)**			
Strongly hesitant	−0·5 (−1·16 to 0·17)	0·34	0·2171
Doubtful	0·93 (0·04 to 1·82)	0·46	0·3015
Willing	−0·28 (−0·60 to 0·04)	0·16	0·7009

Information conditions are outlined in table 1. Scores can range between 7 and 35, with higher scores indicating higher COVID-19 vaccine hesitancy.

Forest plots of the effectiveness of each information condition, compared with the control, moderated by age, gender, income, COVID-19 severity risk, region, and ethnicity, are shown in the appendix (pp 24–32). In these subgroup analyses, a positive mean difference favours condition 1 (control), which can be interpreted as the hesitancy score being higher in the other information conditions. There are few indications of significant moderation, although this information must be interpreted with caution as sample sizes are sometimes small. However, individuals of Black ethnicity tended to have an opposite reaction to some of the information conditions (ie, they had lower hesitancy scores for the control condition) compared with other ethnicities, although this was only significant for condition 3 (collective benefit of not transmitting; mean difference 1·25, 95% CI 0·03 to 2·47; p_interaction_=0·033). Condition 9 (collective and personal benefit) was the only other condition to show significant differences by ethnicity, with Asian individuals showing the greatest reduction in hesitancy (−1·28, –2·26 to –0·31; p_interaction_=0·038). Condition 5 (personal benefit) also had a greater impact for men (−0·53, –0·91 to –0·14) than for women (p_interaction_=0·019).

No participant contacted the lead author or ethics committee about any concerns about the study, which was taken as an indication of the absence of adverse events.

In a post-hoc analysis, the outcome analyses were repeated for only the 14 483 participants who had not been vaccinated). The same significant and non-significant findings were found, with three exceptions: in the strongly hesitant group, significant reductions in hesitancy were also observed with condition 3 (collective benefit of not transmitting the virus; mean difference –0·77, –1·44 to –0·11, adjusted p=0·0373), condition 4 (both collective benefits; –0·98, –1·64 to –0·32, adjusted p=0·0088), and condition 8 (indirectly addressing concerns about vaccine safety related to speed of development; –1·00, –1·68 to –0·33, adjusted p=0·0088) in comparison with the control condition. Personal benefit information was still associated with the greatest lowering of hesitancy (−1·76, –2·42 to –1·09, adjusted p=0·0002), which was significantly greater than the collective benefit conditions (appendix pp 40–48).

The results from our sensitivity analyses using multiple imputation for missing data were comparable to those in the main analysis, showing that excluding the “don't know” responses did not affect our findings (appendix pp 37–39).

## Discussion

Since the start of the COVID-19 vaccination programme in the UK in December, 2020, hesitancy rates in the population appear to have substantially declined. The most notable shift has been towards people wanting to get the COVID-19 vaccination as soon as possible. Back in October, 2020, half the population wanted to get the vaccination as soon as possible; by the time of our study in February, 2021, that figure was closer to three quarters. This is greatly encouraging, although it is highly likely that there will be fluctuations depending on the content of news. A consequence for the study was the need to enrich the large representative participant cohort with individuals selected for COVID-19 vaccine hesitancy. This enrichment preserved the study power to test effectiveness of information conditions by initial vaccine hesitancy levels. As anticipated, this was important because reactions to the information conditions were affected by pre-existing hesitancy levels. The brief information provided, above that of a simple statement of efficacy and safety, did not alter hesitancy levels in those who were willing to be vaccinated or were doubtful (ie, would delay being vaccinated or did not know whether they would get the vaccine). However, there was an impact for people who were strongly hesitant about a COVID-19 vaccine (ie, would avoid getting it for as long as possible or never get it). Individuals who were strongly hesitant were less likely to see the collective benefit of vaccination and had greater safety concerns relating to the speed of development. The hesitancy levels of these individuals were reduced to a small degree by information that highlighted the personal benefits of vaccination or directly addressed speed of development concerns. It was clear that highlighting personal benefit was more effective than emphasising collective benefit for those who were strongly hesitant. It was also worse to combine personal and collective benefit for this group than to present personal benefits alone. Whereas a large majority of the population see the collective benefit and are willing to be vaccinated, simple presentation of the collective benefit rationale is less persuasive for the minority who have not previously accepted it. For this subgroup, it might be harder to shift perspective now to the collective benefits, but highlighting the personal perspective could have a greater attraction. In essence, belief in personal risk from vaccines might be best counterbalanced by personal-benefit messaging. Messaging that reduces hesitancy for this group did not deter the rest of the population. As COVID-19 in all its variant forms is unlikely to vanish, it will probably be necessary to vaccinate the population on a regular and continuing basis. A high-stakes communications challenge on vaccination benefits will confront us for many years to come.

Overall, this study shows that brief, carefully crafted information can alter the willingness to be vaccinated for COVID-19 of those most strongly hesitant. The information could be used in online webpages or when contacting people to make vaccination appointments. However, this study showed no impact of messaging for people who were more mildly hesitant about vaccination or those already willing. Further work is needed to develop messaging that would be persuasive for the important group of people who are currently doubtful. Our findings also indicate that gender and ethnicity, particularly Black ethnicity, might moderate the impact of information provision; this study was, however, underpowered to detect differences by particular ethnic groups. It is also reasonable to suppose that views of who is providing the information (in this instance, views of University of Oxford researchers) might affect the impact of information, which was not tested in the study. Health-care services are typically the most trusted source for vaccination information.^16^ Our messaging text was written with the possibility in mind that it could be used in standard guidance from public health sources and could also be shared and communicated via different sources (eg, local community leaders). Our aim was to provide templates that could be reinforced through distribution and sharing. The study was only designed to detect which broad themes might reduce hesitancy, and could not determine whether smaller linguistic differences between the texts (eg, use of particular words or pronouns) could explain the different outcomes. A future line of research could, for example, unpack different elements involved in the personal benefits thematic construct. The study certainly does not rule out that there could be more successful ways of presenting written information about any of the themes explored. The absence of detection of mediation by COVID-19 vaccine views leaves a further gap in understanding of the significant effects. Written information provision is only one aspect of encouraging vaccine acceptance, and, as this study shows, potentially has only a small effect. It is unknown how important this change might be. However, given the very large number of people who receive this form of communication, even small effects might be important. Future research could assess whether the impact of these messages increases, remains stable, or declines with repetition. The impact of different messages might also vary during the course of a vaccination programme. Written information could be even more powerful when combined with images, animations, videos, or reflective forms of engagement. This could be tested in future large-scale randomised controlled tests. Under certain conditions, written information from institutional sources is also likely to be of benefit when reinforced by information provided by trusted peers and acquaintances via face-to-face conversations, social media, and private messaging apps.^17^

A limitation of this study is that expressed willingness to be vaccinated is unlikely to match exactly actual vaccination behaviour. A further limitation is that it is unknown whether the results will generalise more widely to the UK or to other countries. We used a non-probability online quota sampling method to recruit the majority of the participant group, which, while better than a convenience sample, will still have introduced bias to who was approached to take part. The recruitment of the additional vaccine-hesitant group will have skewed representativeness further. The success of the UK vaccination programme up to the point of the study, and generally high levels of vaccine acceptance in the UK, might mean the findings are of less relevance in other countries. In particular, the prevalence estimates of hesitancy must be treated with caution. The results of the randomised controlled tests of the information conditions can, however, be treated with much greater confidence.

OCEANS-III provides broad initial conclusions about COVID-19 vaccine information provision in the UK but there are numerous issues (eg, detailed dissection of messaging, impact in different groups, source of information provision) that require detailed research attention. Developing such an evidence base about vaccination communication could be immensely important to the success of vaccination programmes.

## Data sharing

De-identified participant data will be available in anonymised form from the corresponding author (DF) on reasonable request (including a study outline) and with an appropriate data sharing agreement, subject to review, following the publication of results. The data analytic plan and full statistical report are available in the appendix (pp 2–53) .

## Declaration of interests

AJP is Chair of UK Department Health and Social Care's Joint Committee on Vaccination & Immunisation, but does not participate in discussions on COVID-19 vaccines, and is a member of the WHO's SAGE. Oxford University has entered into a partnership with AstraZeneca for the development of a coronavirus vaccine. All other authors declare no competing interests.
