# Supplementary material for: Effects of different types of written vaccination information on COVID-19 vaccine hesitancy in the UK (OCEANS-III): a single-blind, parallel-group, randomised controlled trial
Source: Lancet Public Health. 2021 May 13;6(6):e416–27. doi: 10.1016/S2468-2667(21)00096-7 (PMC8116130; doi:10.1016/S2468-2667(21)00096-7)
Supplement: Supplementary appendix [file mmc1.pdf]

THE LANCET
Public Health

Supplementary appendix

This appendix formed part of the original submission and has been peer reviewed. We post it as supplied by the authors.

Supplement to: Freeman D, Loe BS, Yu L-M, et al. Effects of different types of written vaccination information on COVID-19 vaccine hesitancy in the UK (OCEANS-III): a single-blind, parallel-group, randomised controlled trial. *Lancet Public Health* 2021; published online May 12. http://dx.doi.org/10.1016/S2468-2667(21)00096-7.

Appendix

Table of contents

Statistical analysis report pages 2-53
Information conditions pages 54-59

Title: Covid-19 vaccination views: Oxford Coronavirus Explanations, Attitudes, and Narratives Survey (OCEANS III)

STATISTICAL ANALYSIS REPORT
Version 0.5
08/28/2021

Written by: Bao Sheng Loe and Milensu Shanyinde
Reviewed by Trial Statistician: Ly-Mee Yu
Reviewed by Chief Investigator: Daniel Freeman

Version History

Version:	Version Date:	Changes:
0.1	19/03/2021	-
0.2	22/03/2021	Included mediation analysis results
0.3	05/04/2021	Included post-hoc analysis as requested by reviewer. Included additional tables that present the estimated marginal means and mean differences based on randomised conditions as requested by reviewer. Edited typos. Included standard error for all estimated means and rounded all p-values to 4 decimal places (3 decimal places for mediation analysis). Adjusted subgroup analyses to include vaccine hesitancy level at baseline.
0.4	08/04/2021	Updated total n in moderation plots.
0.5	28/04/2021	Updated n in consort diagram

Table of Contents

1. INTRODUCTION *5*

1.1 VALIDATION **6**

1.2 SOFTWARE EMPLOYED **6**

2 METHODS *6*

2.1 BACKGROUND INFORMATION **6**

2.2 TRIAL DESIGN **6**

2.3 OBJECTIVES **6**

Primary Objectives 6

Secondary Objectives 7

2.4 TARGET POPULATION **7**

2.5 INTERVENTIONS **7**

2.6 OUTCOME MEASURES **7**

2.6.1 PRIMARY OUTCOME **7**

2.7 SAMPLE SIZE **8**

2.8 RANDOMISATION AND BLINDING IN THE ANALYSIS STAGE **8**

2.9 DATA CLEANING **8**

2.10 ANALYSIS FOR DATA MONTIORING COMMITTEE MEETINGS **9**

2.11 DEFINTION OF POPULATION FOR ANALYSIS **9**

2.12 DEVIATION FROM SAP **9**

3 RESULTS *9*

3.1 REPRESENTATIVENESS OF STUDY SAMPLE AND PARTICIPANT THROUGHPUT **9**

3.2 RECRUITMENT **9**

3.4 NUMBERS ANALYSED **9**

3.5 PRIMARY OUTCOME **14**

3.5.1 Vaccine hesitancy 14

3.6 SECONDARY OUTCOME **20**

SUBGROUP ANALYSES **24**

MEDIATION ANALYSIS **33**

3.7 SENSITIVTY ANALYSES **37**

3.7.1 MISSING DATA MECHANIMS **37**

4 POST-HOC ANALYSIS *40*

4.1 PRIMARY OUTCOME **40**

4.1.1 Vaccine hesitancy 40

4.2 SECONDARY OUTCOME **45**

5. REFERENCES *49*

6. APPENDICES *51*

List of Tables

Table S1. Baseline characteristics 11
Table S2. Estimated marginal means in each randomised condition 15
Table S3. Mean difference between marginal means across randomised conditions 15
Table S4. Estimated marginal means in each randomised condition across the vaccine hesitancy groups 16
Table S5. Mean difference between marginal means across randomised conditions 17
Table S6. Mean difference between marginal means across randomised conditions 18
Table S7. Interaction model between randomised conditions and vaccine hesitancy groups. 19
Table S8. Mean difference between marginal means across randomised conditions 20
Table S9. Mean difference between marginal means across randomised conditions 21
Table S10. Mean difference between marginal means across randomised conditions 22
Table S11. Mean difference between marginal means across randomised conditions 23
Table S12. Mediation with SEM model for primary outcomes 34
Table S13. Mediation with SEM model for secondary outcomes 36
Table S14. Estimated marginal means in each condition across the vaccine hesitancy groups using multiple imputed data 37
Table S15. Mean difference between marginal means across randomised conditions using multiple imputed data 39
Table S16. Estimated marginal means in each randomised condition 41
Table S17. Mean difference between marginal means across randomised condition 41
Table S18. Estimated marginal means in each randomised condition across the vaccine hesitancy groups have not been vaccinated 42
Table S19. Mean difference between marginal means across randomised conditions 43
Table S20. Mean difference between marginal means across randomised conditions 44
Table S21. Mean difference between marginal means across randomised conditions 45
Table S22. Mean difference between marginal means across randomised conditions 46
Table S23. Mean difference between marginal means across randomised conditions 47
Table S24. Mean difference between marginal means across randomised conditions 48
Table S25. Beliefs about COVID-19 vaccination (Oxford Vaccine Confidence and Complacency Scale) by hesitancy stratification level. 53

List of Figures

Figure S1. Histogram of residuals from linear regression model 14
Figure S2. Subgroup analysis of Oxford Covid-19 Vaccine Hesitancy Score (Condition 2 vs Condition 1) 24
Figure S3. Subgroup analysis of Oxford Covid-19 Vaccine Hesitancy Score (Condition 3 vs Condition 1) 25
Figure S4. Subgroup analysis of Oxford Covid-19 Vaccine Hesitancy Score (Condition 4 vs Condition 1) 26
Figure S5. Subgroup analysis of Oxford Covid-19 Vaccine Hesitancy Score (Condition 5 vs Condition 1) 27
Figure S6. Subgroup analysis of Oxford Covid-19 Vaccine Hesitancy Score (Condition 6 vs Condition 1) 28
Figure S7. Subgroup analysis of Oxford Covid-19 Vaccine Hesitancy Score (Condition 7 vs Condition 1) 29

Figure S8. Subgroup analysis of Oxford Covid-19 Vaccine Hesitancy Score (Condition 8 vs Condition 1) 30
Figure S9. Subgroup analysis of Oxford Covid-19 Vaccine Hesitancy Score (Condition 9 vs Condition 1) 31
Figure S10. Subgroup analysis of Oxford Covid-19 Vaccine Hesitancy Score (Condition 10 vs Condition 1) 32
Figure S11. Mediation with structural equation model for primary outcomes 35
Figure S12. Mediation with structural equation model for secondary outcomes 36
Figure S13. Histogram of residuals from linear regression model 40

1. INTRODUCTION

This document details the analysis set out in **the statistical analysis plan (SAP) V0.4.pdf** dated **19 February 2021** for the OCEAN III randomised study to evaluate the effect of different information provision on willingness to be vaccinated. Subsequent analyses of a more exploratory nature will not be restricted to the methodology set out in the SAP, though they are expected to follow the broad principles described in the SAP.

The SAP will be made available when the principal papers are submitted for publication in a journal. Suggestions for further analysis by journal editors or reviewers will be considered carefully and carried out insofar as to the principles of the analysis strategy. The source of any suggestions, if reported, will be acknowledged.

This report is based on the **Statistical Analysis Plan – V0.4 pdf** dated **19 February 2021.** Any deviation from the statistical analysis plan will be described and justified in the trial report.

Study statistician(s):
Bao Sheng Loe, The Psychometrics Centre, University of Cambridge
a.loe@jbs.cam.ac.uk

Milensu Shanyinde, Nuffield Department of Primary Health Care Sciences, University of Oxford
milensu.shanyinde@phc.ox.ac.uk

Ly-Mee Yu, Nuffield Department of Primary Health Care Sciences, University of Oxford
ly-mee.yu@phc.ox.ac.uk

Chief Investigator:
Professor Daniel Freeman, Department of Psychiatry, University of Oxford
daniel.freeman@psych.ox.ac.uk, 01865 226490

Project Manager
Sinéad Lambe, Department of Psychiatry, University of Oxford
sinead.lambe@psych.ox.ac.uk, 01865 226490

1.1 VALIDATION
Validation of results presented In this report was conducted in R and Stata. Results from R and Stata output were checked for transcription errors.

1.2 SOFTWARE EMPLOYED
Analyses completed in R version 4.0.0. Emmeans R package version 1.5.2.1. Mice R package version 3.9.0. Lavaan R package version 0.6.7. semTools R package version 0.5.3. Analyses verified in Stata version SE 16.1.

2 METHODS

2.1 BACKGROUND INFORMATION

2.2 TRIAL DESIGN
OCEANS III is a single blind parallel groups randomised controlled design. A sample of 18,855 online participants will be recruited for this study. Participants will provide informed consent, complete an item for stratification for vaccine hesitancy level, provide socio-demographic information, randomised (1:1) to receive different vaccine information, and then complete measures of COVID-19 vaccine hesitancy and COVID-19 complacency and confidence beliefs.

Date of start of recruitment:	19th January 2021
Number recruited:	18,855
Date of end of recruitment:	18th February 2021
Target number of subjects:	Originally 15,000, before amendment to increase size

Timing of trial procedures is provided in Appendix I.

2.3 OBJECTIVES
Primary Objectives
The outcome questions are:

1. Does adding information about; the collective benefit of vaccination from not getting ill, the collective benefit of vaccination from not spreading the virus, the personal benefit of getting vaccinated, the seriousness of the SARS-CoV-2, or why speed of development is not a problem (directly and indirectly), lead to lower levels of COVID-19 vaccine hesitancy than a simple statement that vaccination is efficacious and safe? [This is a test against condition 1 (control) of conditions 2, 3, 4, 5, 6, 7, 8.]
2. Does combining collective and personal benefits or combining collective and personal benefits with the seriousness of the virus and indirectly why the speed of development is not a problem lead to lower levels of COVID-19 vaccine hesitancy than a simple statement that vaccination is efficacious and safe? [This is a test against condition 1 (control) of conditions 9 and 10.]

The moderation question is:

1. Is the effect of information provision on COVID-19 vaccine hesitancy moderated by the three groupings of level of hesitancy (positive about vaccination, very unsure, strongly hesitant)?

Secondary Objectives

The outcome questions are:

1. Is emphasising collective benefit (i.e. leads to lower hesitancy) better than emphasising personal benefit? [This is a test of conditions 2 and 3 against 5].
2. Is emphasising why speed of development is not a problem better done directly or indirectly? [This is a test of condition 7 against 8.]
3. Is combining personal and collective benefits better than emphasising personal or collective benefits alone? [This is a test of condition 9 against conditions 4 and 5].
4. Is combining collective and personal benefits with the seriousness of the virus and indirectly why the speed of development is not a problem better than just combining collective and personal benefits? [This is a test of condition 10 against 9.]

The moderation question is:

1. Is the effect of information provision on COVID-19 vaccine hesitancy moderated by age, gender, ethnicity, income, region, or level of Covid-19 health risk?

The mediation question is:

1. If a significant relationship exists between randomised conditions and vaccine hesitancy, can that relationship be explained by COVID-19 vaccine views (the potential collective benefit, the likelihood of COVID-19 infection and the effectiveness of a vaccine, its side-effects, and concerns about the speed of vaccine development)?

2.4 TARGET POPULATION

Inclusion criteria

- Age ≥ 18

Exclusion Criteria

- None

2.5 INTERVENTIONS

2.6 OUTCOME MEASURES

Oxford Covid-19 Vaccine Hesitancy Measure (Freeman et al, 2020). This is a seven-item scale. Item specific response options (Saris, Revilla, Krosnick & Shaeffer, 2010), coded from 1 to 5, are used. A 'Don't know' option is also provided, which is excluded from scoring. The Cronbach's alpha is 0.97. Scores can range between 7 and 35, with higher scores indicating higher COVID-19 vaccine hesitancy.

2.6.1 PRIMARY OUTCOME

The overarching question addressed is: is there specific content about COVID-19 vaccination, above a simple statement of efficacy and safety, that may reduce hesitancy and/or consolidate existing positive views? We are most interested in the effects on those in the general population who are very unsure (approximately 16%) or strongly hesitant (approximately 12%) about a COVID-19 vaccination.

We are also interested in finding out whether the reduction in scores is moderated by different levels of vaccine hesitancy (positive about vaccination, very unsure, strongly

hesitant). The vaccine hesitancy is calculated by adding together the scores for the 7 items. Each item ranging from 1 to 5, and the total score range between 0 to 35. Higher scores indicate greater vaccine hesitancy.

2.7 SAMPLE SIZE

Power calculation: This study is powered to detect a change in hesitancy level for each of the three levels of hesitancy level at baseline, i.e., positive, doubtful, and strongly hesitant. From our previous study, we estimated that there are 71.1% in the general population are positive hesitant with a mean (SD) score on the Oxford Hesitancy Scale of 10.8 (3.5); 16.6% doubtful with mean (SD) score 24.2 (5.8); and 11.7% are strongly hesitant with mean (SD) score 30.6 (3.5). Sample sizes of 96 and 254 will be able to detect a 3-point change in the strong and hesitant groups respectively at 90% power and a type I error at 0.5% (2-sided). For the positive groups, a sample size of 822 will be able to detect a 1-point change. This gives a total sample size of 1,172 is required for each condition. We intend to recruit a total of 15,000 participant to the study to adjust for multiple comparisons in the analysis.

This study is single blinded, as the participants are aware of which arm of the trial they are allocated to, but the researcher assessors are blinded to the study arm of the participant.

According to the original protocol, the target was aimed at 15,000 participants. However, halfway through data collection, vaccine hesitancy levels (as assessed by the stratification question) in the participants were lower than anticipated, and therefore we have planned to recruit approximately 3,500 additional participants who score for vaccine hesitancy (using the stratification question). The total sample is therefore likely to be closer to 18,500.

2.8 RANDOMISATION AND BLINDING IN THE ANALYSIS STAGE

Participants will be randomised equally across all conditions, stratified by three levels of vaccine hesitancy (positive, very doubtful, strongly hesitant). After agreeing to take part, participants will complete a single question: If the vaccine was available at my GP surgery I would: 1. Get it as soon as possible/2. Get it when I have time/3. Delay getting it/ 4. Avoid getting it for as long as possible/ 5. Never get it/ 6. Don't know. This item has a strong loading on the vaccine hesitancy latent factor (0.95) from our OCEAN-II study. The item is: The level of vaccine hesitancy is defined as, Positive=1 or 2, Doubtful=3 or 6, strong hesitant=4 or 5.

Participants are required to read their assigned information condition, and therefore they cannot be blinded to the fact they are given information, although they will be unaware of the other conditions. Participants complete the self-report outcome measures online and therefore the research team can be considered as blinded in relation to assessments. It is notable that all data will be collected by Lucid and the research team will not have any contact with research participants and will therefore be unable to bias the allocation or assessments.

2.9 DATA CLEANING

To ensure quality data is recorded at the end of the data collection process, data cleaning is conducted in real-time by the Lucid crowd sourcing platform. A description of the type of respondents removed is in Appendix II.

2.10 ANALYSIS FOR DATA MONTIORING COMMITTEE MEETINGS

The trial does not have a formal data monitoring committee and there are no planned interim analyses.

2.11 DEFINTION OF POPULATION FOR ANALYSIS

All data will be included in the analysis as far as possible. Participants will be analysed in the groups they were allocated.

The primary analysis included participants with complete response of the primary outcome

The sample size for multiple imputation analysis was different due to missing response from auxiliary variables. See Section 3.4 Numbers Analysed for more information.

2.12 DEVIATION FROM SAP

There were no deviations from the SAP.

3 RESULTS

3.1 REPRESENTATIVENESS OF STUDY SAMPLE AND PARTICIPANT THROUGHPUT

Appendix I provides the Consort Flow Diagram of the participants in the study. Table 1 shows baseline characteristics by randomised condition.

3.2 RECRUITMENT

Recruited started in 19th of January 2021 and carried on to the 5th of February 2021, after recruiting 15,014 participants. A protocol amendment was submitted to increase the sample size from 15,000 to 18,855, after it was determined that vaccine hesitancy levels (as assessed by the stratification question) in the participants were lower than anticipated. Therefore, recruitment continued to the 18th of February and we recruited approximately 3,855 additional participants who score for vaccine hesitancy (using the stratification question).

3.4 NUMBERS ANALYSED

Appendix I provides the total number of participants who participated in each of the study arm which were analysed in the primary and secondary analyses. Of the 18,855 participants enrolled (Table 1), (n=2400) who did not complete all items (i.e. used the 'don't know' option) in the Oxford Vaccine Hesitancy Scale were excluded from further analyses. Thus, the primary and secondary analyses included (n=16,455).

In the subgroup analyses, participants with missing data in both the Oxford Vaccine Hesitancy Scale and demographic characteristics were removed from further analyses. Therefore, the subgroup analyses included (n=15,735). Only complete cases were used in both mediation models (n=965, n=1011) to be consistent with the linear regression model.

Additionally, of the 18,855 participants who enrolled, (n=1413) had missing values in the auxiliary variables (gender and Covid-19 health risk levels). These participants were not included in the multiple imputation analysis. Thus (n=17,442) were included in the multiple imputation analysis.

As requested by the reviewer, the primary and secondary outcome analyses were repeated with only participants who have not been vaccinated (n=14,483).

3.3 BASELINE CHARACTERISTICS OF PARTICIPANTS

Table S1. Baseline characteristics

	Total	Control Group	Condition 2	Condition 3	Condition 4	Condition 5	Condition 6	Condition 7	Condition 8	Condition 9	Condition 10
Mean (SD) age, years	43.23 (18.11)	43.6 (18.09)	43.25 (18.16)	43.05 (18.38)	43.3 (17.76)	43.33 (18.22)	43.31 (18.11)	42.91 (18)	43.58 (18.12)	42.78 (18.07)	43.23 (18.18)
Age Band, n(%)											
18-39	8972 (47.6%)	902 (47.8%)	883 (46.8%)	896 (47.5%)	886 (47%)	891 (47.2%)	893 (47.4%)	915 (48.5%)	898 (47.7%)	913 (48.4%)	895 (47.5%)
40-59	5441 (28.9%)	524 (27.8%)	562 (29.8%)	541 (28.7%)	558 (29.6%)	537 (28.5%)	540 (28.6%)	546 (29%)	529 (28.1%)	548 (29.1%)	556 (29.5%)
>60	4442 (23.6%)	460 (24.4%)	440 (23.3%)	450 (23.8%)	442 (23.4%)	458 (24.3%)	452 (24%)	425 (22.5%)	456 (24.2%)	425 (22.5%)	434 (23%)
Gender, n(%)											
Male	8155 (43.3%)	813 (43.1%)	812 (43.1%)	791 (41.9%)	833 (44.2%)	849 (45%)	816 (43.3%)	800 (42.4%)	839 (44.6%)	811 (43%)	791 (42%)
Female	10512 (55.8%)	1051 (55.7%)	1053 (55.9%)	1070 (56.7%)	1034 (54.8%)	1018 (54%)	1057 (56.1%)	1069 (56.7%)	1029 (54.6%)	1057 (56%)	1074 (57%)
Other	86 (0.5%)	11 (0.6%)	8 (0.4%)	10 (0.5%)	10 (0.5%)	4 (0.2%)	6 (0.3%)	8 (0.4%)	7 (0.4%)	9 (0.5%)	13 (0.7%)
Prefer not to say	102 (0.5%)	11 (0.6%)	12 (0.6%)	16 (0.8%)	9 (0.5%)	15 (0.8%)	6 (0.3%)	9 (0.5%)	8 (0.4%)	9 (0.5%)	7 (0.4%)
Educational Level, n(%)											
No qualifications	1252 (6.6%)	126 (6.7%)	106 (5.6%)	124 (6.6%)	121 (6.4%)	130 (6.9%)	122 (6.5%)	115 (6.1%)	158 (8.4%)	136 (7.2%)	114 (6%)
GCSEs grades A*-C (or equivalent)	5256 (27.9%)	552 (29.3%)	507 (26.9%)	543 (28.8%)	508 (26.9%)	527 (27.9%)	500 (26.5%)	544 (28.8%)	496 (26.3%)	547 (29%)	532 (28.2%)
AS Levels (or equivalent)	795 (4.2%)	80 (4.2%)	82 (4.4%)	83 (4.4%)	75 (4%)	82 (4.3%)	101 (5.4%)	86 (4.6%)	66 (3.5%)	68 (3.6%)	72 (3.8%)
A Levels (or equivalent)	5118 (27.1%)	481 (25.5%)	536 (28.4%)	526 (27.9%)	531 (28.2%)	517 (27.4%)	508 (26.9%)	484 (25.7%)	486 (25.8%)	521 (27.6%)	528 (28%)
Certificate of higher education (e.g. BA, BSc, or equivalent)	4810 (25.5%)	471 (25%)	495 (26.3%)	446 (23.6%)	493 (26.1%)	498 (26.4%)	506 (26.8%)	514 (27.3%)	489 (26%)	442 (23.4%)	456 (24.2%)
Post graduate qualifications (e.g. MA, MSc, PhD, DPhil)	1624 (8.6%)	176 (9.3%)	159 (8.4%)	165 (8.7%)	158 (8.4%)	132 (7%)	148 (7.9%)	143 (7.6%)	188 (10%)	172 (9.1%)	183 (9.7%)
Region, n(%)											

	Total	Control Group	Condition 2	Condition 3	Condition 4	Condition 5	Condition 6	Condition 7	Condition 8	Condition 9	Condition 10
North East	820 (4.3%)	91 (4.8%)	91 (4.8%)	106 (5.6%)	77 (4.1%)	96 (5.1%)	85 (4.5%)	64 (3.4%)	84 (4.5%)	63 (3.3%)	63 (3.3%)
North West	2023 (10.7%)	214 (11.3%)	186 (9.9%)	162 (8.6%)	209 (11.1%)	206 (10.9%)	213 (11.3%)	203 (10.8%)	207 (11%)	213 (11.3%)	210 (11.1%)
Yorkshire and the Humber	1488 (7.9%)	144 (7.6%)	141 (7.5%)	181 (9.6%)	138 (7.3%)	148 (7.8%)	136 (7.2%)	150 (8%)	141 (7.5%)	161 (8.5%)	148 (7.9%)
East Midlands	1385 (7.3%)	142 (7.5%)	135 (7.2%)	141 (7.5%)	146 (7.7%)	149 (7.9%)	128 (6.8%)	151 (8%)	134 (7.1%)	116 (6.2%)	143 (7.6%)
West Midlands	1796 (9.5%)	168 (8.9%)	189 (10%)	182 (9.6%)	166 (8.8%)	154 (8.2%)	205 (10.9%)	174 (9.2%)	182 (9.7%)	194 (10.3%)	182 (9.7%)
East	1381 (7.3%)	132 (7%)	143 (7.6%)	130 (6.9%)	135 (7.2%)	141 (7.5%)	130 (6.9%)	131 (6.9%)	150 (8%)	137 (7.3%)	152 (8.1%)
London	2936 (15.6%)	297 (15.7%)	308 (16.3%)	282 (14.9%)	298 (15.8%)	306 (16.2%)	283 (15%)	283 (15%)	280 (14.9%)	311 (16.5%)	288 (15.3%)
South East	2605 (13.8%)	257 (13.6%)	250 (13.3%)	271 (14.4%)	267 (14.2%)	275 (14.6%)	263 (14%)	264 (14%)	262 (13.9%)	247 (13.1%)	249 (13.2%)
South West	1509 (8%)	144 (7.6%)	152 (8.1%)	150 (7.9%)	147 (7.8%)	141 (7.5%)	160 (8.5%)	157 (8.3%)	148 (7.9%)	142 (7.5%)	168 (8.9%)
Wales	892 (4.7%)	92 (4.9%)	86 (4.6%)	80 (4.2%)	96 (5.1%)	78 (4.1%)	96 (5.1%)	84 (4.5%)	95 (5%)	90 (4.8%)	95 (5%)
Scotland	1528 (8.1%)	148 (7.8%)	164 (8.7%)	158 (8.4%)	154 (8.2%)	140 (7.4%)	135 (7.2%)	177 (9.4%)	144 (7.6%)	160 (8.5%)	148 (7.9%)
Northern Ireland	492 (2.6%)	57 (3%)	40 (2.1%)	44 (2.3%)	53 (2.8%)	52 (2.8%)	51 (2.7%)	48 (2.5%)	56 (3%)	52 (2.8%)	39 (2.1%)
Ethnicity, n(%)											
White	15280 (82%)	1532 (82.4%)	1527 (82%)	1498 (80.5%)	1560 (83.8%)	1538 (82.6%)	1544 (82.7%)	1529 (81.9%)	1539 (82.6%)	1509 (81.1%)	1504 (80.8%)
White + other background	1040 (5.6%)	102 (5.5%)	105 (5.6%)	115 (6.2%)	91 (4.9%)	89 (4.8%)	100 (5.4%)	110 (5.9%)	105 (5.6%)	110 (5.9%)	113 (6.1%)
Black	503 (2.7%)	43 (2.3%)	53 (2.8%)	56 (3%)	50 (2.7%)	53 (2.8%)	58 (3.1%)	47 (2.5%)	39 (2.1%)	52 (2.8%)	52 (2.8%)
Asian	209 (1.1%)	21 (1.1%)	24 (1.3%)	25 (1.3%)	22 (1.2%)	18 (1%)	14 (0.8%)	21 (1.1%)	24 (1.3%)	22 (1.2%)	18 (1%)
other mixed background	1372 (7.4%)	147 (7.9%)	132 (7.1%)	144 (7.7%)	118 (6.3%)	135 (7.3%)	133 (7.1%)	138 (7.4%)	133 (7.1%)	144 (7.7%)	148 (8%)
other	220 (1.2%)	15 (0.8%)	22 (1.2%)	24 (1.3%)	20 (1.1%)	28 (1.5%)	17 (0.9%)	22 (1.2%)	23 (1.2%)	23 (1.2%)	26 (1.4%)
Income, n(%)											
<20k	4840 (25.7%)	460 (24.4%)	483 (25.6%)	477 (25.3%)	487 (25.8%)	484 (25.7%)	492 (26.1%)	480 (25.5%)	495 (26.3%)	497 (26.4%)	485 (25.7%)
20k-40k	6007 (31.9%)	629 (33.4%)	594 (31.5%)	615 (32.6%)	603 (32%)	591 (31.3%)	586 (31.1%)	611 (32.4%)	605 (32.1%)	613 (32.5%)	560 (29.7%)
40-60k	3301 (17.5%)	305 (16.2%)	327 (17.3%)	334 (17.7%)	350 (18.6%)	347 (18.4%)	326 (17.3%)	343 (18.2%)	324 (17.2%)	319 (16.9%)	326 (17.3%)
> 60k	3012 (16%)	304 (16.1%)	307 (16.3%)	284 (15.1%)	311 (16.5%)	283 (15%)	320 (17%)	291 (15.4%)	310 (16.5%)	290 (15.4%)	312 (16.6%)
Prefer not to say	1695 (9%)	164 (8.9%)	157 (8.5%)	159 (8.7%)	117 (6.4%)	164 (8.9%)	141 (7.7%)	146 (7.9%)	133 (7.2%)	148 (8.1%)	182 (10%)

	Total	Control Group	Condition 2	Condition 3	Condition 4	Condition 5	Condition 6	Condition 7	Condition 8	Condition 9	Condition 10
Hesitancy Group, n(%)											
Positive	12463 (66.1%)	1248 (66.2%)	1247 (66.2%)	1245 (66%)	1246 (66.1%)	1248 (66.2%)	1245 (66%)	1245 (66%)	1246 (66.2%)	1247 (66.1%)	1246 (66.1%)
Doubtful	2932 (15.6%)	291 (15.4%)	291 (15.4%)	296 (15.7%)	293 (15.5%)	293 (15.5%)	294 (15.6%)	294 (15.6%)	293 (15.6%)	294 (15.6%)	293 (15.5%)
Hesitant	3460 (18.4%)	347 (18.4%)	347 (18.4%)	346 (18.3%)	347 (18.4%)	345 (18.3%)	346 (18.4%)	347 (18.4%)	344 (18.3%)	345 (18.3%)	346 (18.4%)
Covid 19 risk levels, n(%)											
No - Low risk	11121 (59%)	1115 (59.1%)	1110 (58.9%)	1114 (59%)	1106 (58.6%)	1110 (58.9%)	1138 (60.4%)	1117 (59.2%)	1103 (58.6%)	1091 (57.8%)	1117 (59.3%)
Yes - Moderate risk	5204 (27.6%)	497 (26.4%)	522 (27.7%)	537 (28.5%)	523 (27.7%)	519 (27.5%)	497 (26.4%)	516 (27.4%)	515 (27.3%)	550 (29.2%)	528 (28%)
Yes - High risk	1468 (7.8%)	149 (7.9%)	140 (7.4%)	143 (7.6%)	147 (7.8%)	143 (7.6%)	143 (7.6%)	145 (7.7%)	164 (8.7%)	154 (8.2%)	140 (7.4%)
Do not know	1062 (5.6%)	125 (6.6%)	113 (6%)	93 (4.9%)	110 (5.8%)	114 (6%)	107 (5.7%)	108 (5.7%)	101 (5.4%)	91 (4.8%)	100 (5.3%)

3.5 PRIMARY OUTCOME

3.5.1 Vaccine hesitancy

The primary outcome measure is Oxford Covid-19 Vaccine Hesitancy Measure (Freeman et al, 2020). This is a seven-item scale. Item specific response options (Saris, Revilla, Krosnick & Shaeffer, 2010), coded from 1 to 5, are used. A 'Don't know' option is also provided, which is excluded from scoring. Scores can range between 7 and 35, with higher scores indicating higher COVID-19 vaccine hesitancy.

We checked the assumptions of normality of the model residuals using graphical methods. The outcome residuals were strongly skewed to the right, but the model residuals were found to be sufficiently normally distributed to fit a linear model (Figure S1).

Figure S1. Histogram of residuals from linear regression model

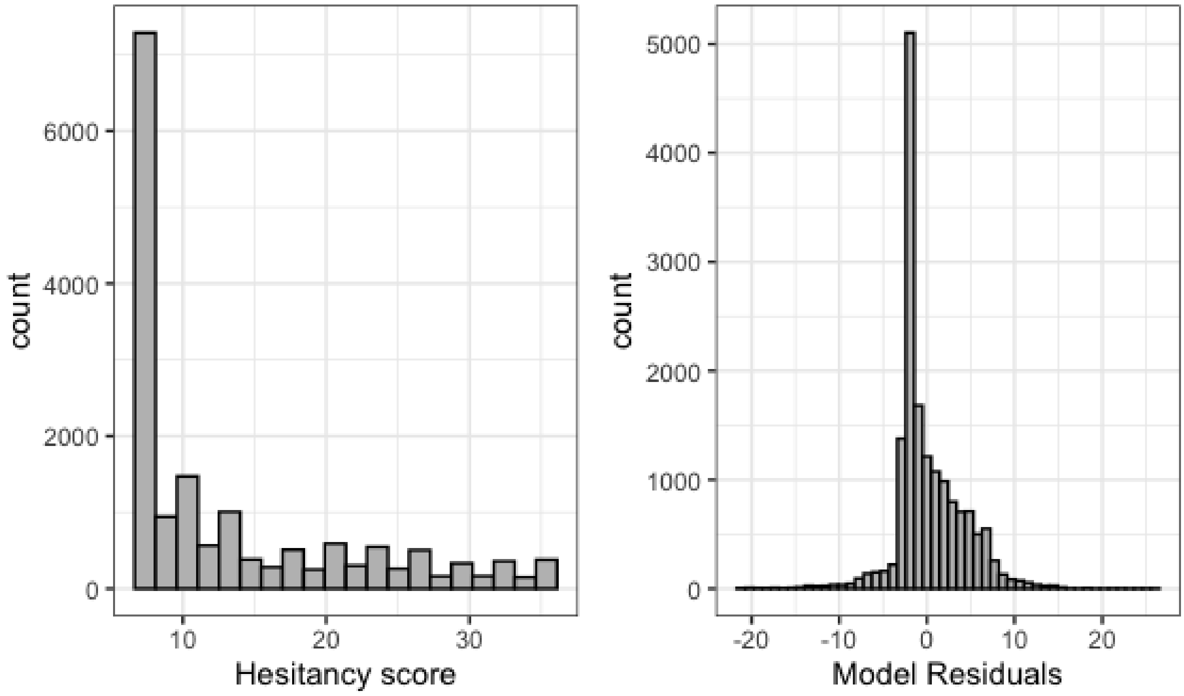

3.5.2 Hesitancy scores between randomised conditions in primary and secondary objectives

The estimated marginal means in each randomised condition and their respective confidence intervals are reported in Table S2.

Table S2. Estimated marginal means in each randomised condition

Randomised Conditions	n	Estimated marginal mean	SE	lower.CI	upper.CI
1	1637	13.61	0.20	13.20	14.00
2	1641	13.52	0.20	13.10	13.90
3	1663	13.63	0.20	13.20	14.00
4	1645	13.48	0.20	13.10	13.90
5	1663	13.31	0.20	12.90	13.70
6	1646	13.53	0.20	13.10	13.90
7	1645	13.43	0.20	13.00	13.80
8	1644	13.40	0.20	13.00	13.80
9	1648	13.71	0.20	13.30	14.10
10	1623	13.21	0.20	12.80	13.60

SE = Standard error

There were no significant differences across the randomised conditions (Table S3).

Table S3. Mean difference between marginal means across randomised conditions

Contrast	Estimated mean difference (C.I.)	SE	Adjusted P-value*
2 - 1	-0.09 (-0.65; 0.48)	0.29	0.9136
3 - 1	0.02 (-0.54; 0.58)	0.29	0.9561
4 - 1	-0.13 (-0.70; 0.43)	0.29	0.9136
5 - 1	-0.30 (-0.86; 0.26)	0.29	0.8595
5 - 2	-0.22 (-0.78; 0.34)	0.29	0.8595
5 - 3	-0.32 (-0.88; 0.24)	0.29	0.8595
6 - 1	-0.08 (-0.64; 0.49)	0.29	0.9136
7 - 1	-0.18 (-0.75; 0.38)	0.29	0.8697
8 - 1	-0.21 (-0.77; 0.35)	0.29	0.8595
8 - 7	-0.03 (-0.59; 0.53)	0.29	0.9561
9 - 1	0.10 (-0.46; 0.66)	0.29	0.9136
9 - 4	0.23 (-0.33; 0.79)	0.29	0.8595
9 - 5	0.40 (-0.16; 0.96)	0.29	0.8195
10 - 1	-0.40 (-0.96; 0.16)	0.29	0.8195
10 - 9	-0.5 (-1.06; 0.06)	0.29	0.8195

* P values adjustment using False Discovery Rate method. C.I. = Confidence intervals. SE = Standard error

3.5.3 Impact of hesitancy vaccine levels on reduction of hesitancy scores between Condition 1 (Control) vs Conditions 2, 3, 4, 5, 6, 7, 8

The estimated marginal means in each randomised condition across the vaccine hesitancy groups and their respective confidence intervals are reported in Table S4.

Table S4. Estimated marginal means in each randomised condition across the vaccine hesitancy groups

	n	Estimated marginal mean	SE	lower.CI	upper.CI
**Vaccine Hesitancy group - Strongly hesitant**					
**Randomised condition**					
1	268	28.53	0.24	28.05	29.01
2	269	28.01	0.24	27.54	28.49
3	271	28.06	0.24	27.58	28.53
4	279	27.85	0.24	27.38	28.32
5	270	27.04	0.24	26.57	27.51
6	263	28.29	0.25	27.81	28.76
7	261	27.62	0.25	27.14	28.11
8	257	27.79	0.25	27.30	28.28
9	284	28.17	0.24	27.70	28.63
10	264	27.67	0.24	27.19	28.15
**Vaccine Hesitancy group - Doubtful**					
**Randomised condition**					
1	160	20.49	0.31	19.87	21.10
2	157	20.71	0.32	20.09	21.33
3	181	20.51	0.30	19.93	21.09
4	167	20.13	0.31	19.53	20.73
5	169	20.79	0.31	20.19	21.39
6	170	20.08	0.30	19.49	20.68
7	170	20.60	0.30	20.00	21.20
8	171	20.88	0.30	20.29	21.48
9	163	19.87	0.31	19.26	20.48
10	143	20.80	0.33	20.15	21.45
**Vaccine Hesitancy group - Willing**					
**Randomised condition**					
1	1209	9.39	0.11	9.17	9.62
2	1215	9.39	0.11	9.16	9.61
3	1211	9.37	0.11	9.14	9.59

4	1199	9.21	0.12	8.98	9.43
5	1224	9.24	0.11	9.02	9.47
6	1213	9.42	0.11	9.20	9.64
7	1214	9.37	0.11	9.15	9.59
8	1216	9.30	0.11	9.08	9.53
9	1201	9.46	0.12	9.23	9.68
10	1216	9.18	0.11	8.95	9.40

SE = Standard error

There were significant differences between groups (Condition 5 vs 1) and (Condition 7 vs 1) in the strongly hesitancy group. Participants randomised to condition 5 showed significant reduced levels of hesitancy (mean differences (95% C.I.): -1.49 (-2.16;-0.82); adjusted p-value < 0.0001) compared to condition 1. Participants randomised to condition 7 showed significant reduced levels of hesitancy (mean differences (95% C.I.): -0.91 (-1.58; -0.23); adjusted p-value =0.0261) compared to condition 1 (Table S5).

There were no other significant differences in the strongly hesitant group and in the other vaccine hesitancy groups across the randomised conditions [Condition 1 (control) vs Conditions 2, 3, 4, 5, 6, 7, 8].

Table S5. Mean difference between marginal means across randomised conditions

	**Contrast**	**Estimated mean difference (C.I.)**	**SE**	**Adjusted P-value***
**Vaccine Hesitancy group -Strongly hesitant**				
**Randomised condition**				
	2 - 1	-0.51 (-1.19; 0.16)	0.34	0.2171
	3 - 1	-0.47 (-1.14; 0.20)	0.34	0.2254
	4 - 1	-0.68 (-1.35; -0.01)	0.34	0.0846
	5 - 1	-1.49 (-2.16; -0.82)	0.34	0.0015
	6 - 1	-0.24 (-0.92; 0.43)	0.35	0.5117
	7 - 1	-0.91 (-1.58; -0.23)	0.35	0.0261
	8 - 1	-0.74 (-1.42; -0.06)	0.35	0.0703
**Vaccine Hesitancy group - Doubtful**				
**Randomised condition**				
	2 - 1	0.23 (-0.65; 1.10)	0.45	0.7656
	3 - 1	0.02 (-0.82; 0.87)	0.43	0.9615
	4 - 1	-0.36 (-1.22; 0.51)	0.44	0.7515
	5 - 1	0.31 (-0.55; 1.16)	0.44	0.7515
	6 - 1	-0.41 (-1.26; 0.45)	0.44	0.7515
	7 - 1	0.11 (-0.74; 0.97)	0.44	0.9178
	8 - 1	0.40 (-0.46; 1.25)	0.44	0.7515
**Vaccine Hesitancy group - Willing**				

	Contrast	Estimated mean difference (C.I.)	SE	Adjusted P-value*
**Randomised condition**				
	2 - 1	-0.01 (-0.32; 0.31)	0.16	0.9741
	3 - 1	-0.02 (-0.34; 0.29)	0.16	0.9497
	4 - 1	-0.19 (-0.50; 0.13)	0.16	0.7443
	5 - 1	-0.15 (-0.46; 0.17)	0.16	0.7924
	6 - 1	0.03 (-0.29; 0.34)	0.16	0.9497
	7 - 1	-0.02 (-0.34; 0.29)	0.16	0.9497
	8 - 1	-0.09 (-0.41; 0.23)	0.16	0.9477

* P values adjustment using False Discovery Rate method. C.I. = Confidence intervals. SE = Standard error

3.5.4 Impact of hesitancy vaccine levels on reduction of hesitancy scores between Condition 1 (Control) vs Conditions 9 and 10

There was a significant difference between groups (Condition 10 vs 1) in the strongly hesitancy group. Participants randomised to condition 10 showed significant reduced levels of hesitancy (mean differences (95% C.I.): -0.86 (-1.53; -0.18); adjusted p-value = 0.0313) compared to condition 1 (Table S6).

There were no other significant differences in the strongly hesitant group and in the other vaccine hesitancy groups across the randomised conditions [Condition 1 (control) vs Conditions 9 and 10].

Table S6. Mean difference between marginal means across randomised conditions

	Contrast	Estimated mean difference (C.I.)	SE	Adjusted P-value*
**Vaccine Hesitancy group -Strongly hesitant**				
**Randomised condition**				
	9 - 1	-0.36 (-1.03; 0.30)	0.34	0.3514
	10 - 1	-0.86 (-1.53; -0.18)	0.34	0.0313
**Vaccine Hesitancy group - Doubtful**				
**Randomised condition**				
	9 - 1	-0.62 (-1.48; 0.25)	0.44	0.7515
	10 - 1	0.32 (-0.58; 1.21)	0.46	0.7515
**Vaccine Hesitancy group - Willing**				
**Randomised condition**				
	9 - 1	0.06 (-0.25; 0.38)	0.16	0.9477
	10 - 1	-0.22 (-0.53; 0.10)	0.16	0.7009

* P values adjustment using False Discovery Rate method. C.I. = Confidence intervals. SE = Standard error

3.5.5 MODERATION ANALYSIS

Table S7. Interaction model between randomised conditions and vaccine hesitancy groups

Term	Estimated mean difference	SE	lower.CI	upper.CI	P-value
condition 2 vs 1	0.23	0.45	-0.65	1.10	0.6125
condition 3 vs 1	0.02	0.43	-0.82	0.87	0.9615
condition 4 vs 1	-0.36	0.44	-1.22	0.50	0.4179
condition 5 vs 1	0.31	0.44	-0.55	1.16	0.4855
condition 6 vs 1	-0.41	0.44	-1.26	0.45	0.3542
condition 7 vs 1	0.11	0.44	-0.74	0.97	0.7970
condition 8 vs 1	0.40	0.44	-0.46	1.25	0.3650
condition 9 vs 1	-0.62	0.44	-1.48	0.25	0.1630
condition 10 vs 1	0.32	0.46	-0.58	1.21	0.4882
groupPositive	-11.09	0.33	-11.75	-10.44	<0.001
groupHesitant	8.04	0.40	7.26	8.82	<0.001
2vs1:groupPositive	-0.23	0.47	-1.16	0.70	0.6260
3vs1:groupPositive	-0.05	0.46	-0.95	0.86	0.9214
4vs1:groupPositive	0.17	0.47	-0.75	1.09	0.7182
5vs1:groupPositive	-0.45	0.47	-1.37	0.46	0.3296
6vs1:groupPositive	0.43	0.47	-0.48	1.34	0.3550
7vs1:groupPositive	-0.14	0.47	-1.05	0.78	0.7712
8vs1:groupPositive	-0.48	0.47	-1.40	0.43	0.2974
9vs1:groupPositive	0.68	0.47	-0.24	1.60	0.1485
10vs1:groupPositive	-0.53	0.48	-1.48	0.42	0.2722
2vs1:groupHesitant	-0.74	0.56	-1.84	0.36	0.1877
3vs1:groupHesitant	-0.50	0.55	-1.57	0.58	0.3679
4vs1:groupHesitant	-0.32	0.56	-1.41	0.76	0.5587
5vs1:groupHesitant	-1.79	0.56	-2.88	-0.71	0.0013
6vs1:groupHesitant	0.16	0.56	-0.93	1.25	0.7732
7vs1:groupHesitant	-1.02	0.56	-2.11	0.07	0.0677
8vs1:groupHesitant	-1.14	0.56	-2.23	-0.04	0.0417
9vs1:groupHesitant	0.25	0.56	-0.84	1.34	0.6506
10vs1:groupHesitant	-1.18	0.57	-2.30	-0.05	0.0398

SE = Standard error

3.6 SECONDARY OUTCOME

3.6.1 Impact of hesitancy vaccine levels on reduction of hesitancy scores between Condition 2 vs Conditions 3 and 5

There were significant differences between groups (Condition 5 vs 2) and (Condition 5 vs 3) in the strongly hesitancy group. Participants randomised to condition 5 showed significant reduced levels of hesitancy (mean differences (95% C.I.): -0.97 (-1.64; -0.30); adjusted p-value = 0.0165) compared to condition 2. Participants randomised to condition 5 showed significant reduced levels of hesitancy (mean differences (95% C.I.): -1.01 (-1.68; -0.35); adjusted p-value = 0.0150) compared to condition 3 (Table S8).

There were no other significant differences in the other vaccine hesitancy groups across the randomised conditions [Condition 5 vs Conditions 2 and 3].

Table S8. Mean difference between marginal means across randomised conditions

	Contrast	Estimated mean difference (C.I.)	SE	Adjusted P-value*
**Vaccine Hesitancy group -Strongly hesitant**				
**Randomised condition**				
	5 - 2	-0.97 (-1.64; -0.30)	0.34	0.0165
	5 - 3	-1.01 (-1.68; -0.35)	0.34	0.0150
**Vaccine Hesitancy group – Doubtful**				
**Randomised condition**				
	5 - 2	0.08 (-0.78; 0.94)	0.44	0.9178
	5 - 3	0.28 (-0.55; 1.12)	0.43	0.7515
**Vaccine Hesitancy group – Willing**				
**Randomised condition**				
	5 - 2	-0.14 (-0.46; 0.17)	0.16	0.7924
	5 - 3	-0.12 (-0.44; 0.19)	0.16	0.8211

* P values adjustment using False Discovery Rate method. C.I. = Confidence intervals. SE = Standard error

3.6.2 Impact of hesitancy vaccine levels on reduction of hesitancy scores between Condition 7 and Conditions 8

There were no significant differences in the vaccine hesitancy groups across the randomised conditions [Condition 7 vs Condition 8] (Table S9).

Table S9. Mean difference between marginal means across randomised conditions

	Contrast	Estimated mean difference (C.I.)	SE	Adjusted P-value*
**Vaccine Hesitancy group -Strongly hesitant**				
**Randomised condition**				
	8 - 7	-0.17 (-0.52; 0.85)	0.35	0.6355
**Vaccine Hesitancy group – Doubtful**				
**Randomised condition**				
	8 - 7	0.28 (-0.56; 1.13)	0.43	0.7515
**Vaccine Hesitancy group – Willing**				
**Randomised condition**				
	8 - 7	-0.07 (-0.38; 0.25)	0.16	0.9477

* P values adjustment using False Discovery Rate method. C.I. = Confidence intervals. SE = Standard error

3.6.3 Impact of hesitancy vaccine levels on reduction of hesitancy scores between Condition 9 vs Conditions 4 and 5

There was a significant difference between groups (Condition 9 vs 5) in the strongly hesitancy group. Participants randomised to condition 9 showed significant increased levels of hesitancy (mean differences (95% C.I.): 1.12 (0.46; 1.79); adjusted p-value = 0.0068) compared to condition 5 (Table S10).

There were no other significant differences in the strongly hesitant group and in the other vaccine hesitancy groups across the randomised conditions [Condition 9 vs conditions 4 and 5].

Table S10. Mean difference between marginal means across randomised conditions

	Contrast	Estimated mean difference (C.I.)	SE	Adjusted P-value*
**Vaccine Hesitancy group -Strongly hesitant**				
**Randomised condition**				
	9 - 4	0.32 (-0.34; 0.97)	0.34	0.3980
	9 - 5	1.12 (0.46; 1.79)	0.34	0.0068
**Vaccine Hesitancy group – Doubtful**				
**Randomised condition**				
	9 - 4	-0.26 (-1.12; 0.60)	0.44	0.7515
	9 - 5	-0.92 (-1.78; -0.07)	0.44	0.3015
**Vaccine Hesitancy group – Willing**				
**Randomised condition**				
	9 - 4	0.25 (-0.07; 0.57)	0.16	0.7009
	9 - 5	0.21 (-0.10; 0.53)	0.16	0.7009

* P values adjustment using False Discovery Rate method. C.I. = Confidence intervals. SE = Standard error

3.6.4 Impact of hesitancy vaccine levels on reduction of hesitancy scores between Condition 9 and Condition 10

There were no significant differences in the vaccine hesitancy groups across the randomised conditions [Condition 10 vs condition 9] (Table S11).

Table S11. Mean difference between marginal means across randomised conditions

	Contrast	Estimated mean difference (C.I.)	SE	Adjusted P-value*
**Vaccine Hesitancy group -Strongly hesitant**				
**Randomised condition**				
	10 - 9	-0.50 (-1.16; 0.17)	0.34	0.2171
**Vaccine Hesitancy group – Doubtful**				
**Randomised condition**				
	10 - 9	0.93 (0.04; 1.82)	0.46	0.3015
**Vaccine Hesitancy group – Willing**				
**Randomised condition**				
	10 - 9	-0.28 (-0.60; 0.04)	0.16	0.7009

* P values adjustment using False Discovery Rate method. C.I. = Confidence intervals. SE = Standard error

SUBGROUP ANALYSES

Differences observed within subgroups should be interpreted with caution as sample sizes are small.

Figure S2. Subgroup analysis of Oxford Covid-19 Vaccine Hesitancy Score (Condition 2 vs Condition 1)

Variable and Subgroup	Condition 2 (n=1561)	Condition 1 (n=1544)	Mean difference (95% CI)	P-value*
**Age**				0·74
18-39	669	686	-0·05 (-0·46, 0·35)	
40-59	482	441	0·11 (-0·38, 0·60)	
>60	410	417	-0·17 (-0·69, 0·34)	
**Gender**				0·67
Male	711	718	-0·02 (-0·41, 0·38)	
Female	845	822	-0·05 (-0·42, 0·31)	
**Income**				0·34
<20k	392	348	0·27 (-0·28, 0·82)	
20-40k	505	557	-0·14 (-0·60, 0·31)	
40-60k	289	258	-0·02 (-0·65, 0·62)	
>60k	276	276	0·04 (-0·59, 0·67)	
Prefer not to say	99	105	-0·97 (-2·01, 0·07)	
**Risk**				0·84
Low	944	955	0·03 (-0·31, 0·37)	
Moderate	486	452	-0·13 (-0·61, 0·36)	
High	131	137	-0·16 (-1·07, 0·74)	
**Region**				0·067
North East	77	64	-1·32 (-2·58, -0·06)	
North West	159	179	0·31 (-0·50, 1·12)	
Yorkshire and the Humber	127	122	0·15 (-0·79, 1·09)	
East Midlands	107	109	-1·33 (-2·34, -0·32)	
West Midlands	139	130	-0·84 (-1·75, 0·07)	
East	125	116	0·50 (-0·46, 1·46)	
London	250	222	0·51 (-0·18, 1·19)	
South East	202	222	0·06 (-0·66, 0·79)	
South West	133	123	-0·13 (-1·06, 0·80)	
Wales	73	82	-0·24 (-1·44, 0·95)	
Scotland	138	124	0·27 (-0·64, 1·19)	
Northern Island	31	51	0·39 (-1·30, 2·08)	
**Ethnicity**				0·44
White	1316	1305	0·04 (-0·25, 0·33)	
Black	68	67	0·24 (-1·04, 1·52)	
White other backgroud	39	36	-0·35 (-2·07, 1·36)	
Other mixed background	16	16	-0·61 (-3·24, 2·01)	
Asian	104	112	-1·05 (-2·06, -0·04)	
Other	18	8	0·66 (-2·50, 3·81)	

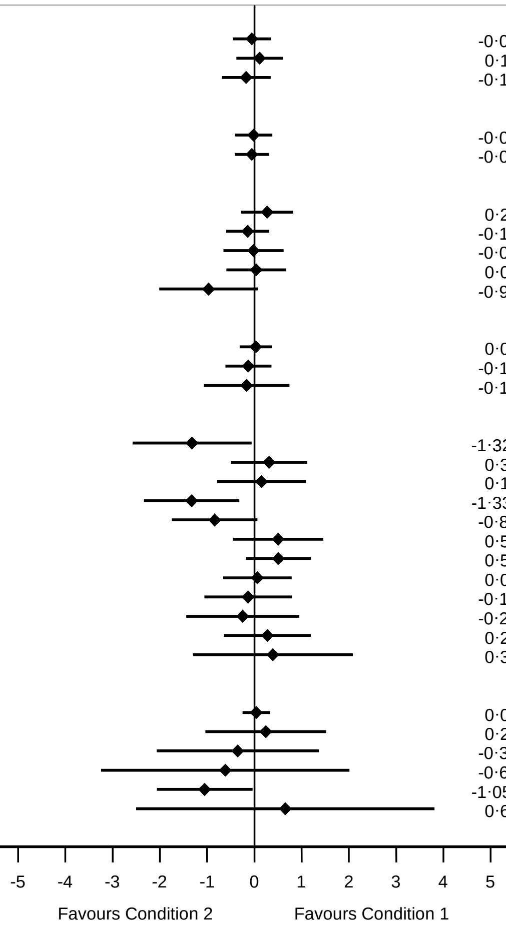

*Test for interaction

Figure S3. Subgroup analysis of Oxford Covid-19 Vaccine Hesitancy Score (Condition 3 vs Condition 1)

Variable and Subgroup	Condition 3 (n=1596)	Condition 1 (n=1544)	Mean difference (95% CI)	P-value*
**Age**				0·99
18-39	708	686	-0·11 (-0·51, 0·28)	
40-59	466	441	-0·13 (-0·63, 0·36)	
>60	422	417	-0·16 (-0·67, 0·36)	
**Gender**				0·20
Male	703	718	0·06 (-0·34, 0·45)	
Female	885	822	-0·27 (-0·63, 0·09)	
**Income**				0·54
<20k	387	348	-0·06 (-0·61, 0·48)	
20-40k	541	557	-0·25 (-0·70, 0·20)	
40-60k	303	258	-0·29 (-0·92, 0·34)	
>60k	252	276	0·34 (-0·30, 0·99)	
Prefer not to say	113	105	-0·49 (-1·50, 0·51)	
**Risk**				0·86
Low	976	955	-0·17 (-0·51, 0·17)	
Moderate	491	452	-0·02 (-0·50, 0·47)	
High	129	137	-0·24 (-1·15, 0·68)	
**Region**				0·62
North East	90	64	-0·92 (-2·13, 0·30)	
North West	134	179	0·38 (-0·47, 1·23)	
Yorkshire and the Humber	155	122	-0·03 (-0·93, 0·87)	
East Midlands	122	109	-1·20 (-2·18, -0·22)	
West Midlands	158	130	-0·36 (-1·24, 0·52)	
East	116	116	-0·17 (-1·15, 0·80)	
London	219	222	0·04 (-0·67, 0·75)	
South East	236	222	-0·03 (-0·73, 0·66)	
South West	130	123	-0·16 (-1·09, 0·78)	
Wales	63	82	-0·18 (-1·42, 1·07)	
Scotland	137	124	0·15 (-0·77, 1·07)	
Northern Island	36	51	0·44 (-1·18, 2·06)	
**Ethnicity**				0·033
White	1306	1305	-0·07 (-0·37, 0·22)	
Black	83	67	1·25 (0·03, 2·47)	
White other backgroud	48	36	-1·71 (-3·35, -0·08)	
Other mixed background	21	16	-0·70 (-3·16, 1·77)	
Asian	118	112	-1·03 (-2·00, -0·05)	
Other	20	8	0·35 (-2·76, 3·45)	

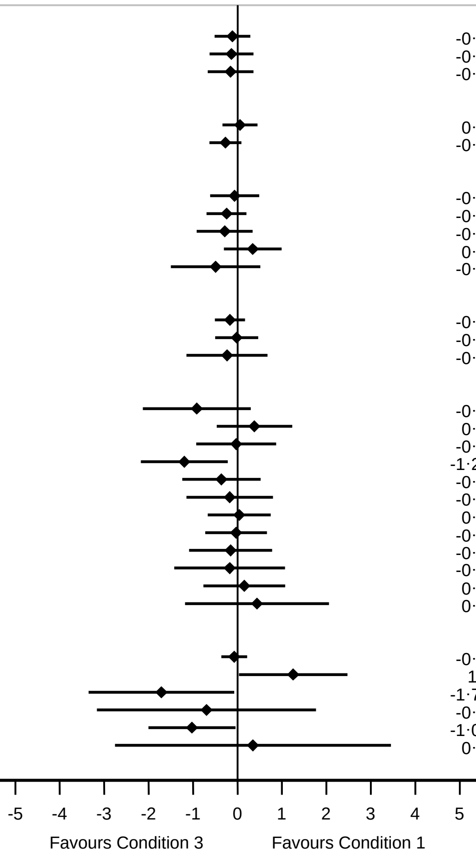

*Test for interaction

Figure S4. Subgroup analysis of Oxford Covid-19 Vaccine Hesitancy Score (Condition 4 vs Condition 1)

Variable and Subgroup	Condition 4 (n=1579)	Condition 1 (n=1544)	Mean difference (95% CI)	P-value*
**Age**				0·17
18-39	686	686	-0·49 (-0·89, -0·09)	
40-59	491	441	0·08 (-0·40, 0·57)	
>60	402	417	-0·08 (-0·60, 0·44)	
**Gender**				0·24
Male	731	718	0·01 (-0·38, 0·40)	
Female	841	822	-0·40 (-0·76, -0·03)	
**Income**				0·54
<20k	386	348	-0·27 (-0·82, 0·27)	
20-40k	521	557	-0·16 (-0·61, 0·30)	
40-60k	313	258	-0·57 (-1·20, 0·05)	
>60k	284	276	0·21 (-0·42, 0·84)	
Prefer not to say	75	105	-0·25 (-1·37, 0·88)	
**Risk**				0·052
Low	967	955	-0·38 (-0·72, -0·05)	
Moderate	482	452	0·27 (-0·22, 0·76)	
High	130	137	-0·70 (-1·61, 0·21)	
**Region**				0·36
North East	73	64	-0·55 (-1·82, 0·72)	
North West	180	179	-0·34 (-1·12, 0·44)	
Yorkshire and the Humber	120	122	0·36 (-0·59, 1·32)	
East Midlands	120	109	-1·03 (-2·02, -0·05)	
West Midlands	137	130	-0·78 (-1·69, 0·13)	
East	111	116	0·54 (-0·44, 1·53)	
London	228	222	0·17 (-0·53, 0·87)	
South East	218	222	-0·59 (-1·30, 0·11)	
South West	128	123	-0·37 (-1·31, 0·56)	
Wales	83	82	0·06 (-1·10, 1·21)	
Scotland	134	124	-0·16 (-1·08, 0·77)	
Northern Island	47	51	0·64 (-0·86, 2·14)	
**Ethnicity**				0·63
White	1335	1305	-0·17 (-0·46, 0·12)	
Black	70	67	0·20 (-1·07, 1·46)	
White other backgroud	42	36	-1·28 (-2·96, 0·41)	
Other mixed background	19	16	-0·76 (-3·28, 1·77)	
Asian	97	112	-0·58 (-1·61, 0·45)	
Other	16	8	1·27 (-1·95, 4·48)	

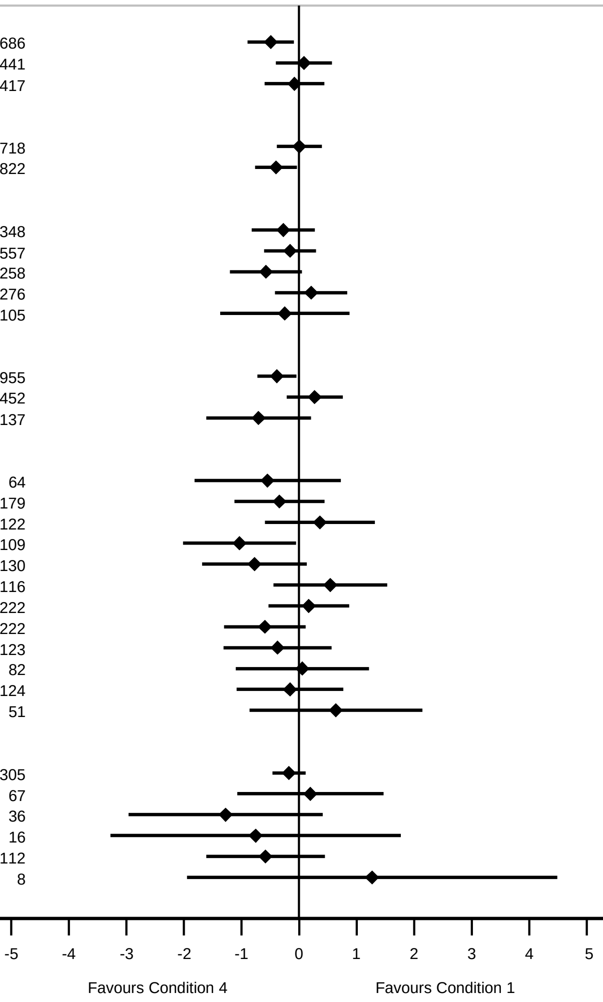

* Test for interaction

Figure S5. Subgroup analysis of Oxford Covid-19 Vaccine Hesitancy Score (Condition 5 vs Condition 1)

Variable and Subgroup	Condition 5 (n=1576)	Condition 1 (n= 1544)	Mean difference (95% CI)	P-value*
**Age**				0·34
18-39	677	686	-0·52 (-0·92, -0·12)	
40-59	464	441	-0·15 (-0·65, 0·34)	
>60	435	417	-0·10 (-0·61, 0·41)	
**Gender**				0·019
Male	736	718	-0·53 (-0·91, -0·14)	
Female	837	822	-0·07 (-0·44, 0·29)	
**Income**				0·65
<20k	382	348	0·04 (-0·51, 0·59)	
20-40k	512	557	-0·41 (-0·87, 0·04)	
40-60k	312	258	-0·36 (-0·99, 0·26)	
>60k	258	276	-0·31 (-0·96, 0·33)	
Prefer not to say	112	105	-0·75 (-1·75, 0·26)	
**Risk**				0·48
Low	967	955	-0·32 (-0·66, 0·02)	
Moderate	480	452	-0·12 (-0·60, 0·37)	
High	129	137	-0·75 (-1·66, 0·16)	
**Region**				0·47
North East	78	64	-0·99 (-2·24, 0·27)	
North West	166	179	0·07 (-0·73, 0·87)	
Yorkshire and the Humber	128	122	0·27 (-0·67, 1·21)	
East Midlands	129	109	-0·87 (-1·83, 0·10)	
West Midlands	132	130	-1·15 (-2·07, -0·24)	
East	124	116	-0·00 (-0·96, 0·96)	
London	233	222	-0·37 (-1·07, 0·32)	
South East	243	222	-0·12 (-0·81, 0·57)	
South West	129	123	-0·68 (-1·61, 0·26)	
Wales	67	82	-0·24 (-1·47, 0·98)	
Scotland	105	124	-0·09 (-1·08, 0·89)	
Northern Island	42	51	0·56 (-0·99, 2·10)	
**Ethnicity**				0·84
White	1334	1305	-0·26 (-0·55, 0·03)	
Black	63	67	-0·07 (-1·38, 1·23)	
White other backgroud	45	36	-0·20 (-1·86, 1·46)	
Other mixed background	11	16	0·38 (-2·53, 3·29)	
Asian	103	112	-0·97 (-1·98, 0·04)	
Other	20	8	-0·20 (-3·31, 2·91)	

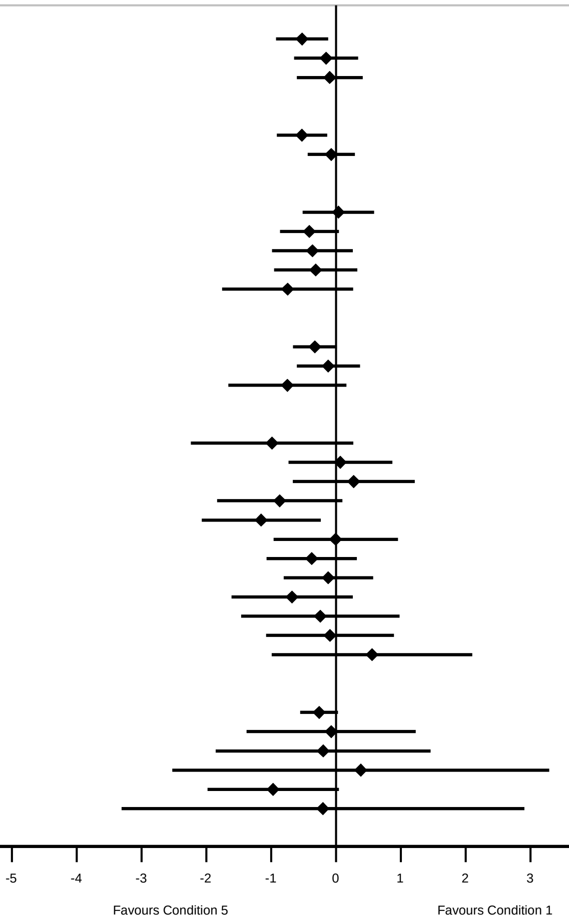

*Test for interaction

Figure S6. Subgroup analysis of Oxford Covid-19 Vaccine Hesitancy Score (Condition 6 vs Condition 1)

Variable and Subgroup	Condition 6 (n=1581)	Condition 1 (n= 1544)	Mean difference (95% CI)	P-value*
**Age**				0·32
18-39	703	686	-0·29 (-0·69, 0·11)	
40-59	458	441	0·20 (-0·30, 0·69)	
>60	420	417	-0·09 (-0·61, 0·42)	
**Gender**				0·25
Male	727	718	-0·10 (-0·49, 0·29)	
Female	850	822	-0·07 (-0·44, 0·29)	
**Income**				0·97
<20k	386	348	-0·03 (-0·57, 0·52)	
20-40k	522	557	-0·03 (-0·48, 0·42)	
40-60k	296	258	-0·09 (-0·73, 0·54)	
>60k	285	276	-0·30 (-0·93, 0·33)	
Prefer not to say	92	105	-0·08 (-1·14, 0·98)	
**Risk**				0·94
Low	985	955	-0·12 (-0·45, 0·22)	
Moderate	462	452	-0·03 (-0·52, 0·46)	
High	134	137	-0·19 (-1·09, 0·71)	
**Region**				0·26
North East	72	64	-1·56 (-2·83, -0·28)	
North West	180	179	0·06 (-0·73, 0·84)	
Yorkshire and the Humber	111	122	-0·00 (-0·98, 0·97)	
East Midlands	105	109	-0·91 (-1·92, 0·11)	
West Midlands	165	130	0·05 (-0·82, 0·92)	
East	111	116	0·23 (-0·76, 1·21)	
London	227	222	0·32 (-0·38, 1·02)	
South East	224	222	0·01 (-0·70, 0·71)	
South West	143	123	-0·74 (-1·65, 0·17)	
Wales	88	82	-0·09 (-1·23, 1·05)	
Scotland	113	124	0·46 (-0·50, 1·43)	
Northern Island	42	51	-0·81 (-2·36, 0·74)	
**Ethnicity**				0·44
White	1332	1305	-0·13 (-0·42, 0·16)	
Black	67	67	1·02 (-0·26, 2·31)	
White other backgroud	55	36	-0·31 (-1·90, 1·29)	
Other mixed background	12	16	-1·45 (-4·28, 1·39)	
Asian	105	112	0·04 (-0·97, 1·05)	
Other	10	8	-1·75 (-5·27, 1·77)	

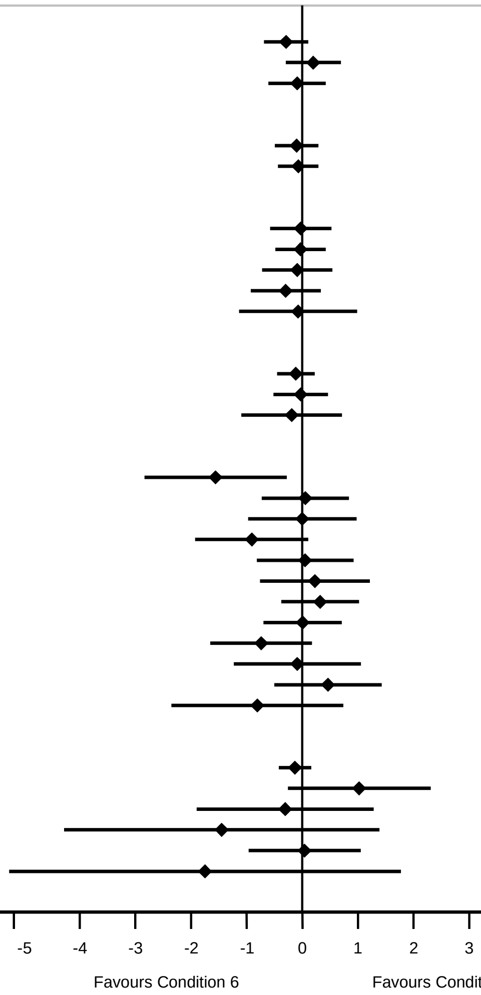

*Test for interaction

Figure S7. Subgroup analysis of Oxford Covid-19 Vaccine Hesitancy Score (Condition 7 vs Condition 1)

Variable and Subgroup	Condition 7 (n=1578)	Condition 1 (n= 1544)	Mean difference (95% CI)	P-value*
**Age**				0·25
18-39	707	686	-0·39 (-0·79, 0·00)	
40-59	488	441	0·14 (-0·35, 0·63)	
>60	383	417	-0·11 (-0·64, 0·41)	
**Gender**				0·57
Male	720	718	-0·14 (-0·53, 0·25)	
Female	854	822	-0·17 (-0·54, 0·19)	
**Income**				0·68
<20k	371	348	0·11 (-0·44, 0·67)	
20-40k	533	557	-0·23 (-0·68, 0·22)	
40-60k	307	258	-0·21 (-0·83, 0·42)	
>60k	267	276	-0·14 (-0·78, 0·50)	
Prefer not to say	100	105	-0·75 (-1·79, 0·29)	
**Risk**				0·61
Low	968	955	-0·25 (-0·59, 0·09)	
Moderate	478	452	0·05 (-0·44, 0·53)	
High	132	137	-0·23 (-1·14, 0·67)	
**Region**				0·50
North East	56	64	-0·51 (-1·87, 0·85)	
North West	167	179	0·48 (-0·32, 1·28)	
Yorkshire and the Humber	117	122	0·51 (-0·46, 1·47)	
East Midlands	129	109	-0·59 (-1·55, 0·38)	
West Midlands	146	130	-0·45 (-1·34, 0·45)	
East	113	116	-0·04 (-1·02, 0·94)	
London	222	222	-0·27 (-0·97, 0·44)	
South East	225	222	-0·43 (-1·14, 0·27)	
South West	139	123	-0·79 (-1·71, 0·13)	
Wales	79	82	-0·57 (-1·74, 0·60)	
Scotland	147	124	0·39 (-0·52, 1·29)	
Northern Island	38	51	0·24 (-1·35, 1·83)	
**Ethnicity**				0·12
White	1318	1305	-0·07 (-0·36, 0·22)	
Black	84	67	0·27 (-0·95, 1·48)	
White other backgroud	35	36	-0·92 (-2·68, 0·85)	
Other mixed background	14	16	-1·10 (-3·82, 1·62)	
Asian	110	112	-1·36 (-2·35, -0·36)	
Other	17	8	1·60 (-1·59, 4·78)	

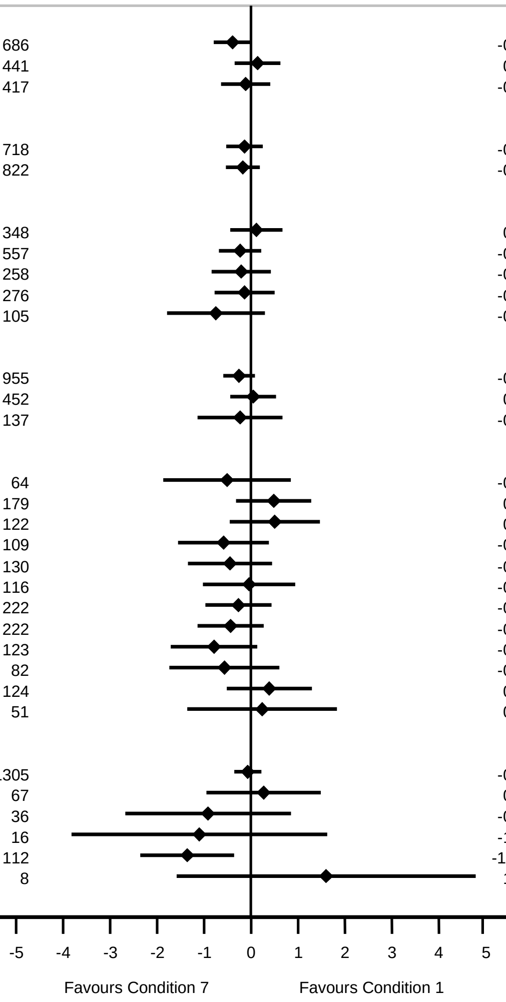

*Test for interaction

Figure S8. Subgroup analysis of Oxford Covid-19 Vaccine Hesitancy Score (Condition 8 vs Condition 1)

Variable and Subgroup	Condition 8 (n=1581)	Condition 1 (n= 1544)	Mean difference (95% CI)	P-value*
**Age**				0·42
18-39	691	686	-0·34 (-0·74, 0·06)	
40-59	463	441	0·05 (-0·45, 0·54)	
>60	427	417	-0·02 (-0·53, 0·49)	
**Gender**				0·27
Male	728	718	0·06 (-0·33, 0·45)	
Female	851	822	-0·30 (-0·67, 0·06)	
**Income**				0·60
<20k	392	348	0·04 (-0·51, 0·59)	
20-40k	519	557	-0·39 (-0·84, 0·07)	
40-60k	293	258	-0·28 (-0·91, 0·36)	
>60k	281	276	0·12 (-0·51, 0·75)	
Prefer not to say	96	105	0·19 (-0·86, 1·24)	
**Risk**				0·92
Low	965	955	-0·10 (-0·44, 0·24)	
Moderate	475	452	-0·22 (-0·71, 0·26)	
High	141	137	-0·16 (-1·05, 0·73)	
**Region**				0·64
North East	70	64	-1·40 (-2·69, -0·12)	
North West	165	179	0·04 (-0·76, 0·84)	
Yorkshire and the Humber	117	122	-0·02 (-0·98, 0·94)	
East Midlands	116	109	-0·05 (-1·05, 0·94)	
West Midlands	154	130	-0·18 (-1·06, 0·71)	
East	129	116	0·47 (-0·49, 1·42)	
London	220	222	0·09 (-0·62, 0·79)	
South East	232	222	-0·32 (-1·02, 0·38)	
South West	129	123	-0·65 (-1·59, 0·28)	
Wales	81	82	-0·60 (-1·76, 0·57)	
Scotland	122	124	-0·13 (-1·07, 0·82)	
Northern Island	46	51	0·56 (-0·95, 2·07)	
**Ethnicity**				0·33
White	1325	1305	-0·14 (-0·43, 0·15)	
Black	80	67	0·48 (-0·75, 1·70)	
White other backgroud	30	36	0·83 (-1·01, 2·66)	
Other mixed background	21	16	-0·56 (-3·02, 1·91)	
Asian	107	112	-0·91 (-1·91, 0·09)	
Other	18	8	1·74 (-1·42, 4·89)	

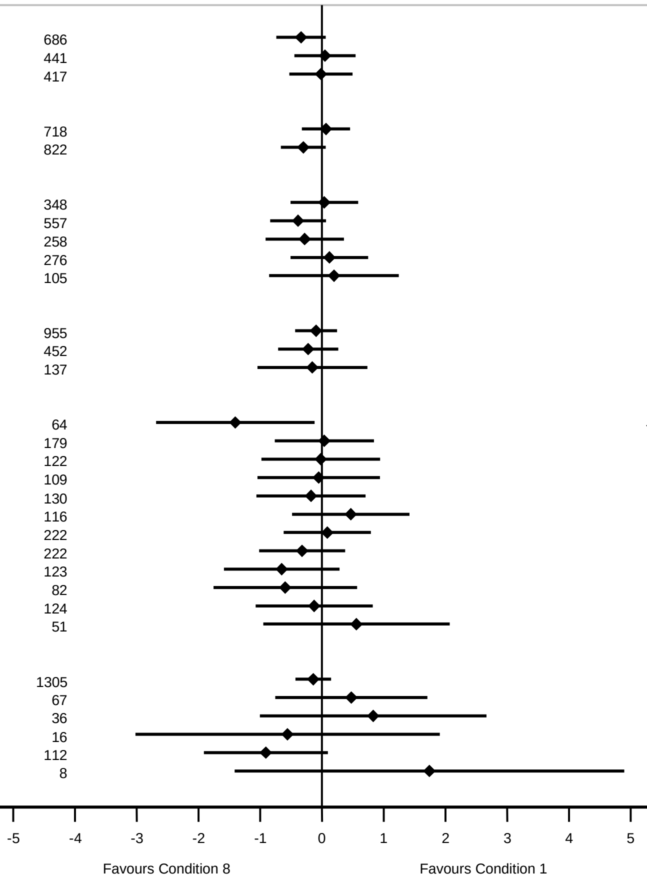

*Test for interaction

Figure S9. Subgroup analysis of Oxford Covid-19 Vaccine Hesitancy Score (Condition 9 vs Condition 1)

Variable and Subgroup	Condition 9 (n=1584)	Condition 1 (n= 1544)	Mean difference (95% CI)	P-value*
**Age**				0·75
18-39	712	686	-0·27 (-0·67, 0·13)	
40-59	475	441	-0·05 (-0·54, 0·44)	
>60	397	417	-0·09 (-0·61, 0·43)	
**Gender**				0·26
Male	702	718	-0·00 (-0·40, 0·39)	
Female	876	822	-0·27 (-0·64, 0·09)	
**Income**				0·80
<20k	397	348	-0·22 (-0·76, 0·33)	
20-40k	532	557	-0·24 (-0·69, 0·21)	
40-60k	278	258	-0·22 (-0·86, 0·42)	
>60k	270	276	0·22 (-0·42, 0·85)	
Prefer not to say	107	105	-0·32 (-1·34, 0·70)	
**Risk**				0·70
Low	948	955	-0·19 (-0·54, 0·15)	
Moderate	497	452	-0·01 (-0·49, 0·47)	
High	139	137	-0·42 (-1·31, 0·48)	
**Region**				0·79
North East	53	64	-0·51 (-1·89, 0·87)	
North West	178	179	-0·00 (-0·79, 0·78)	
Yorkshire and the Humber	135	122	-0·04 (-0·97, 0·89)	
East Midlands	94	109	-0·59 (-1·63, 0·46)	
West Midlands	170	130	-0·41 (-1·28, 0·45)	
East	118	116	-0·11 (-1·08, 0·86)	
London	253	222	0·47 (-0·21, 1·15)	
South East	213	222	-0·35 (-1·06, 0·36)	
South West	122	123	-0·46 (-1·41, 0·49)	
Wales	77	82	-0·35 (-1·52, 0·83)	
Scotland	129	124	-0·47 (-1·40, 0·47)	
Northern Island	42	51	0·72 (-0·83, 2·27)	
**Ethnicity**				0·038
White	1307	1305	-0·07 (-0·36, 0·22)	
Black	75	67	1·08 (-0·17, 2·33)	
White other backgroud	43	36	-1·26 (-2·94, 0·42)	
Other mixed background	22	16	-1·12 (-3·56, 1·33)	
Asian	122	112	-1·28 (-2·26, -0·31)	
Other	15	8	-0·91 (-4·16, 2·34)	

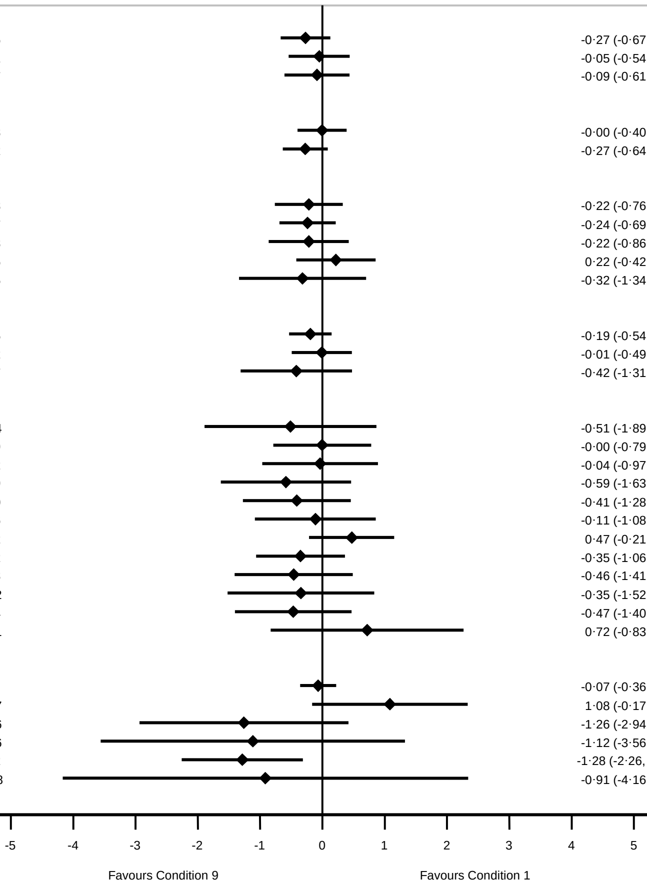

*Test for interaction

Figure S10. Subgroup analysis of Oxford Covid-19 Vaccine Hesitancy Score (Condition 10 vs Condition 1)

Variable and Subgroup	Condition 10 (n=1555)	Condition 1 (n= 1544)	Mean difference (95% CI)	P-value*
**Age**				0·39
18-39	656	686	-0·47 (-0·88, -0·07)	
40-59	493	441	-0·03 (-0·52, 0·46)	
>60	406	417	-0·28 (-0·80, 0·23)	
**Gender**				0·058
Male	708	718	-0·06 (-0·45, 0·34)	
Female	841	822	-0·47 (-0·83, -0·10)	
**Income**				0·99
<20k	384	348	-0·20 (-0·75, 0·35)	
20-40k	488	557	-0·37 (-0·83, 0·09)	
40-60k	279	258	-0·24 (-0·88, 0·40)	
>60k	282	276	-0·28 (-0·91, 0·35)	
Prefer not to say	122	105	-0·36 (-1·35, 0·63)	
**Risk**				0·12
Low	946	955	-0·44 (-0·78, -0·10)	
Moderate	479	452	0·12 (-0·37, 0·61)	
High	130	137	-0·68 (-1·59, 0·23)	
**Region**				0·45
North East	46	64	-0·73 (-2·17, 0·71)	
North West	177	179	-0·56 (-1·35, 0·23)	
Yorkshire and the Humber	123	122	-0·31 (-1·26, 0·64)	
East Midlands	110	109	-0·76 (-1·76, 0·25)	
West Midlands	153	130	-0·45 (-1·34, 0·43)	
East	129	116	-0·25 (-1·21, 0·70)	
London	220	222	0·02 (-0·69, 0·73)	
South East	211	222	-0·55 (-1·27, 0·16)	
South West	152	123	-0·38 (-1·28, 0·52)	
Wales	80	82	-0·28 (-1·45, 0·88)	
Scotland	123	124	0·20 (-0·75, 1·14)	
Northern Island	31	51	1·97 (0·28, 3·66)	
**Ethnicity**				0·26
White	1283	1305	-0·25 (-0·54, 0·04)	
Black	82	67	0·36 (-0·86, 1·59)	
White other backgroud	34	36	-0·60 (-2·38, 1·18)	
Other mixed background	13	16	-0·19 (-2·96, 2·59)	
Asian	121	112	-1·29 (-2·26, -0·31)	
Other	22	8	1·44 (-1·63, 4·50)	

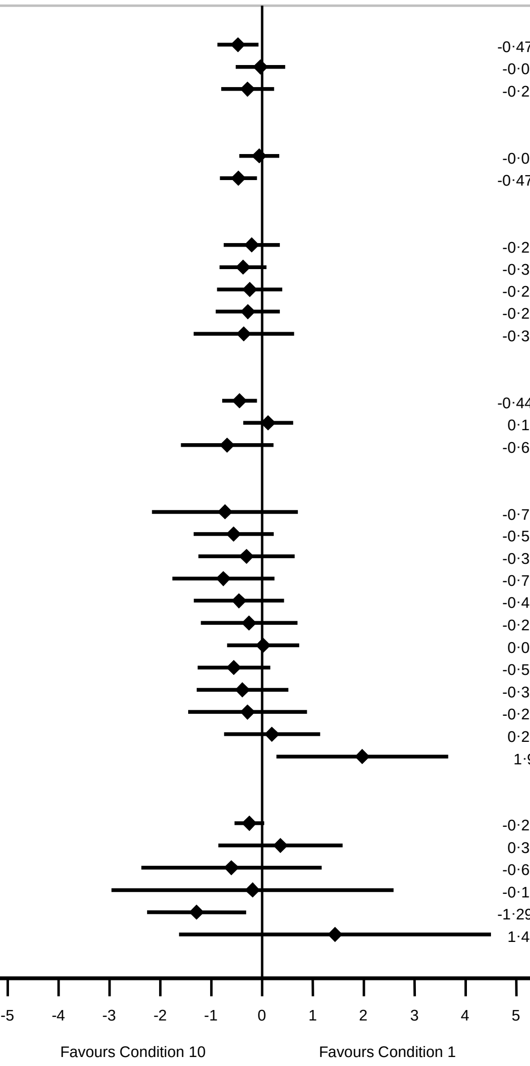

*Test for interaction

MEDIATION ANALYSIS

The results in the primary and secondary outcomes showed that a significant relationship existed between specific randomised conditions and vaccine hesitancy (Tables S5, S6, S8 & S10) in the strongly hesitant group. The primary outcomes that showed significant differences in vaccine hesitancy scores between randomised conditions were (Condition 7 vs Condition 1; Condition 5 vs Condition 1; Condition 10 vs Condition 1). The secondary outcomes that showed significant differences in vaccine hesitancy scores between randomised conditions were (Condition 5 vs Condition 2; Condition 5 vs Condition 3; Condition 5 vs Condition 9). Therefore, the objective of the mediation analysis was to assess whether the changes in the vaccine hesitancy scores for these conditions in the strongly hesitant group were explained by the COVID-19 vaccine views (Beliefs).

Two mediation analyses were performed given that the primary and secondary outcomes used different comparison groups. Condition 1 (control) was used as the reference group in the first mediation model and condition 5 was used as the reference group in the second mediation model. The following mediation analyses were performed according to the SAP version 0.4. dated 19th February 2021. That is, the mediation analyses were carried out under the structural equation modelling framework. Specifically, the factor scores of the Oxford Covid-19 Vaccine Hesitancy Measure will be the response variable with randomised group as the exposure and a higher order latent variable (beliefs) derived from the four subscales of the Oxford Vaccine Confidence and Complacency Scale as indicator will be the mediator variable. Age, gender, ethnicity, income, region and level of Covid-19 health risk were included in the models as controls. However, instead of allowing for missing data, we only used complete cases to be consistent with the linear regression model. The maximum likelihood (ML) estimation procedure with robust (Huber-White) standard errors and a scaled test statistic was employed to estimate the models due to non-normal data. The model's goodness of fit is considered acceptable when the root mean square error of approximate (RMSEA) and standardized root mean square residual (SRMR) are less than 0.08 (Kline, 2005). Additional fit indices included the comparative fit index (CFI) and Tucker-Lewis Index (TLI), all of which should exceed 0.90 (Kline, 2010), with RMSEA < 0.06, and CFI and TLI > 0.95 indicating good model data fit (Hu & Bentler, 1999).

Instead of testing a series of regression equations as proposed by Baron and Kenny (1986) to evaluate mediation, we applied a formal inferential test of the effects in the mediation model as suggested by Hayes & Preacher (2014). We employed the Monte Carlo (MC) method described by MacKinnon, Lockwood & Williams (2004) as it does not require the assumption of normality of the effect's sampling distribution. Using indirect effect as an example, this approach would randomly simulate values based on the parameters of the indirect effect and the associated standard errors, and estimate a confidence interval (CI). We used the proposed replication of 20,000 simulated values to generate the CIs for all the effects in the mediation models. Variables that do not have zero in the CI indicates a significant effect. The advantages of generating CIs for the effects in the mediation model using Monte Carlo are further described in Kristopher, Preacher & Selig (2012). We also applied an omnibus test (Wald test) to the indirect effects to evaluate the extent to which the coefficients are simultaneously

equal to zero (null hypothesis). If the test fails to reject the null hypothesis, then it would indicate that there is no presence of any indirect effect.

Table S12 presents the mediation with SEM model that includes the unstandardised estimates for all relevant parameters for the randomised conditions in the primary outcomes. The sample size was (n=965). The indirect effects did not significantly mediate the paths between the randomised conditions and vaccine hesitancy score. The Wald statistic is 2.55 with df = 3 and p = 0.47, indicating that the null hypothesis was not rejected. The collective results indicated that there was no presence of a mediation effect. The model showed an acceptable model fit according to the SEM fit statistics and indices: robust $\chi^2(df=716) = 1418.87$[1], $p \leq 0.001$; Root Mean Square Error of Approximation (RMSEA)= 0.03; Standardised Root Mean Square Residual (SRMR) = 0.03; Comparative fit index (CFI)= 0.92; Tucker-Lewis index (TLI)= 0.91. The mediation with SEM model diagram with factor loadings and unstandardised effects are shown in Figure S11.

Table S12. Mediation with SEM model for primary outcomes

	Effect	SE	lower CI*	upper CI*	P-value
**Effect of IV (conditions) on mediator (beliefs)**					
Condition 5	-0.05	0.09	-0.23	0.14	0.619
Condition 7	-0.05	0.09	-0.22	0.13	0.603
Condition 10	-0.14	0.09	-0.31	0.04	0.121
**Unique effect of mediator (beliefs) on outcome (hesitancy score)**					
Beliefs	0.76	0.04	0.67	0.85	< 0.001
**Indirect effect (beliefs and conditions)**					
Indirect effect of beliefs and condition 5	-0.04	0.07	-0.17	0.11	0.618
Indirect effect of beliefs and condition 7	-0.04	0.07	-0.17	0.10	0.603
Indirect effect of beliefs and condition 10	-0.11	0.07	-0.24	0.03	0.123
**Direct effect of IV (conditions) on outcome (hesitancy score)**					
Condition 5	-0.23	0.07	-0.36	-0.10	0.001
Condition 7	-0.13	0.07	-0.25	0.00	0.054
Condition 10	-0.05	0.07	-0.17	0.09	0.488
**Total effect**					
Indirect + Direct	-0.58	0.21	-0.99	-0.16	0.006

*Monte Carlo confidence interval. SE = Standard error

[1] The chi-square index is sensitive to sample size. Hence, an alternative method to evaluate model fit is the relative chi-square (x2/df), which is 1.98 in this case. There is no clear consensus of an acceptable ratio, though several researchers have recommended the ratio to be less than 2 to 5 as a general rule of thumb (Wheaton, Muthen, Alwin, & Summers, 1977; March & Hocevar, 1985; Byrne, 1989; Barrett, 2007).

Figure S11. Mediation with structural equation model for primary outcomes

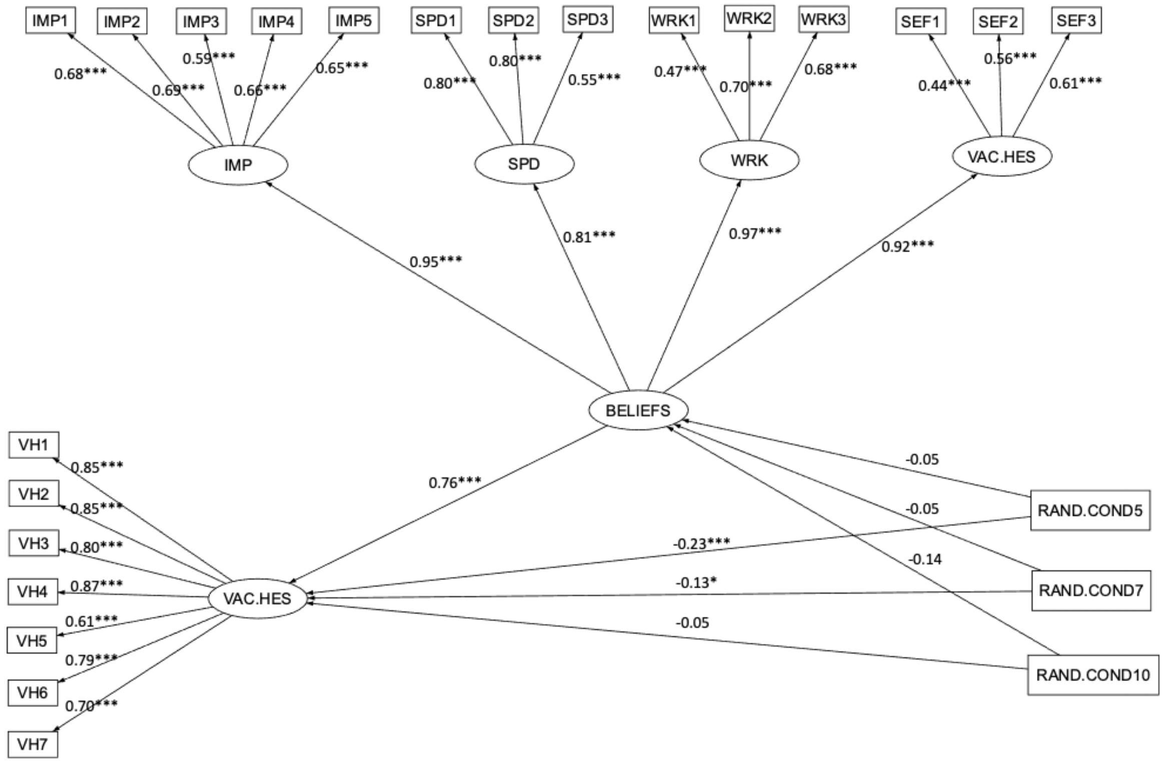

Note: *p < 0.01, **p < 0.01, ***p < 0.001
IMP = collective importance; SPD = speed of development; WRK = vaccine will be effective; S.EF = side effects; VAC.HES = vaccine hesitancy; AND.COND = randomised condition
The direct and indirect effects in the structural model are presented as *unstandardised* estimates. The factor loadings in the measurement model are presented as *standardised* estimates.

Table S13 presents the mediation with SEM model that includes the unstandardised estimates for all relevant parameters for the randomised conditions in the secondary outcomes. The sample size was (n= 1011). Similar to Table 10, the indirect effects did not significantly mediate the paths between the randomised conditions and vaccine hesitancy score. The Wald statistic is 0.44 with df = 3 and p = 0.93, indicating that the null hypothesis was not rejected. The collective results indicated that there was no presence of a mediation effect. The model showed an acceptable model fit according to the SEM fit statistics and indices: robust χ^2(*df*=716) = 1573.58[2], *p*≤0.001; Root Mean Square Error of Approximation (RMSEA)= 0.03; Standardised Root Mean Square Residual (SRMR) = 0.03; Comparative fit index (CFI)= 0.91; Tucker-Lewis index (TLI)= 0.90. The mediation with SEM model diagram with factor loadings and unstandardised effects are shown in Figure S12.

[2] The relative chi-square is 2.20.

Table S13. Mediation with SEM model for secondary outcomes

	Effect	SE	lower CI*	upper CI*	P-value
**Effect of IV (conditions) on mediator (beliefs)**					
Condition 2	0.05	0.09	-0.12	0.23	0.539
Condition 3	0.01	0.09	-0.16	0.19	0.904
Condition 9	0.03	0.09	-0.14	0.20	0.732
**Unique effect of mediator (beliefs) on outcome (hesitancy score)**					
Beliefs	0.78	0.04	0.69	0.86	< 0.001
**Indirect effect (beliefs and conditions)**					
Indirect effect of beliefs and condition 2	0.04	0.07	-0.09	0.18	0.540
Indirect effect of beliefs and condition 3	0.01	0.07	-0.13	0.15	0.904
Indirect effect of beliefs and condition 9	0.02	0.07	-0.11	0.15	0.732
**Direct effect of IV (conditions) on outcome (hesitancy score)**					
Condition 2	0.16	0.06	0.04	0.28	0.011
Condition 3	0.16	0.06	0.04	0.29	0.010
Condition 9	0.19	0.06	0.07	0.32	0.002
**Total effect**					
Indirect + Direct	0.59	0.21	0.17	1.00	0.005

*Monte Carlo confidence interval. SE = Standard error

Figure S12. Mediation with structural equation model for secondary outcomes

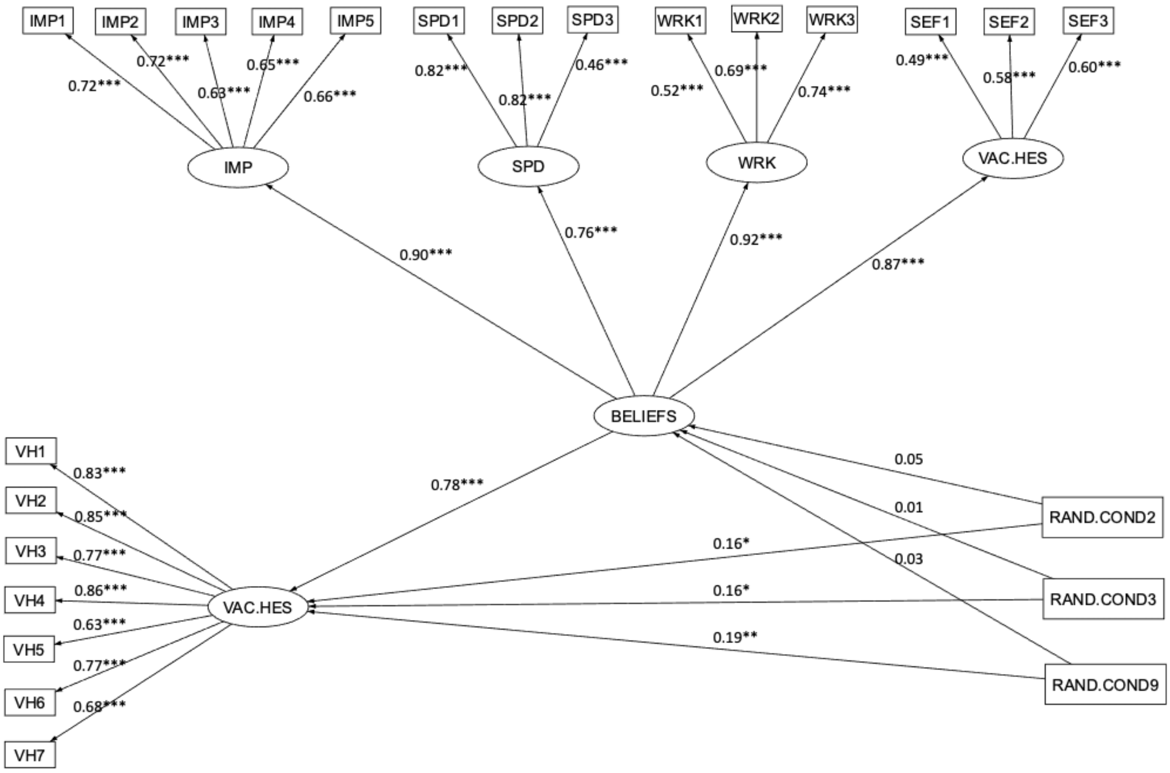

Note: *p < 0.01, **p < 0.01, ***p < 0.001
IMP = collective importance; SPD = speed of development; WRK = vaccine will be effective; S.EF = side effects; VAC.HES = vaccine hesitancy; RAND.COND = randomised condition

The direct and indirect effects in the structural model are presented as *unstandardised* estimates. The factor loadings in the measurement model are presented as *standardised* estimates.

3.7 SENSITIVTY ANALYSES

3.7.1 MISSING DATA MECHANIMS

Missing data measuring vaccine hesitancy at the item level was assumed to be missing at random (MAR) and thus, multiple imputation (MI) was used to impute missing values (Schafer, 1999). Predictive mean matching was used as the imputation algorithm which is appropriate for numeric data. The conditional predictive distribution of the item level responses required to be imputed was adjusted to account for the information from age, gender, and level of Covid-19 health risk, all of which were known to be significant predictors of vaccine hesitancy as described in OCEAN II (Freeman et al., 2020). Participants who answered 50% or more to the 'don't know' option in the Oxford Covid_19 Vaccine Hesitancy measure were removed. We only used complete cases for age, gender and level of covid-19 health risk. Participants that had missing values in either of the three variables were also removed. Hence, the sample size for MI was n=17,442.

MI was imputed 50 times at the item level, and the responses from each imputed dataset were summed to create total hesitancy scores. The regression model was estimated 50 times and the predictions were averaged based on Rubin's rules (Rubin, 1987) to calculate the estimated marginal means. Tables S14 and S15 shows the marginal means and the marginal mean differences between the conditions with imputed data respectively. The results from these tables were found to be comparable to those that used complete cases in the primary outcomes (Tables S4, S5 and S6). That is, only participants that were randomised to conditions 5, 7 and 10 in the strongly hesitant group showed significant reduced levels of hesitancy compared to condition 1 (control) (Table S14).

Table S14. Estimated marginal means in each condition across the vaccine hesitancy groups using multiple imputed data

	n	Estimated marginal mean	SE	lower.CI	upper.CI
**Vaccine Hesitancy group - Strongly hesitant**					
**Randomised condition**					
1	301	28.23	0.23	27.78	28.69
2	311	27.71	0.23	27.27	28.16
3	315	27.89	0.23	27.44	28.33
4	311	27.74	0.23	27.29	28.19
5	306	26.96	0.23	26.51	27.42
6	306	27.74	0.23	27.28	28.19
7	317	27.23	0.23	26.79	27.68
8	308	27.82	0.23	27.37	28.27
9	319	27.96	0.23	27.51	28.40
10	308	27.32	0.23	26.87	27.77

**Vaccine Hesitancy group - Doubtful**						
**Randomised condition**						
	1	227	20.91	0.27	20.39	21.43
	2	225	21.09	0.27	20.56	21.62
	3	233	20.59	0.27	20.07	21.11
	4	224	20.32	0.27	19.79	20.85
	5	229	21.21	0.27	20.69	21.74
	6	234	20.26	0.26	19.74	20.78
	7	225	20.71	0.27	20.18	21.24
	8	244	21.06	0.26	20.55	21.57
	9	223	20.30	0.27	19.76	20.83
	10	221	20.86	0.27	20.32	21.39
**Vaccine Hesitancy group - Willing**						
**Randomised condition**						
	1	1198	9.51	0.12	9.29	9.74
	2	1204	9.52	0.12	9.29	9.74
	3	1206	9.43	0.12	9.20	9.65
	4	1204	9.36	0.12	9.14	9.59
	5	1203	9.30	0.12	9.07	9.53
	6	1202	9.49	0.12	9.27	9.72
	7	1210	9.44	0.12	9.21	9.66
	8	1206	9.39	0.12	9.16	9.61
	9	1210	9.57	0.12	9.34	9.79
	10	1212	9.27	0.12	9.04	9.49

SE = Standard error

Table S15. Mean difference between marginal means across randomised conditions using multiple imputed data

	Contrast	Estimated mean difference (C.I.)	SE	Adjusted P-value*
**Vaccine Hesitancy group -Strongly hesitant**				
**Randomised condition**				
	2 - 1	-0.52 (-1.16; 0.12)	0.33	0.1986
	3 - 1	-0.34 (-0.98; 0.30)	0.33	0.3297
	4 - 1	-0.49 (-1.13; 0.15)	0.33	0.1986
	5 - 1	-1.27 (-1.91; -0.63)	0.33	0.0009
	6 - 1	-0.49 (-1.14; 0.15)	0.33	0.1986
	7 - 1	-1.00 (-1.63; -0.36)	0.33	0.0099
	8 - 1	-0.41 (-1.05; 0.23)	0.33	0.2737
	9 - 1	-0.28 (-0.91; 0.36)	0.32	0.3952
	10 - 1	-0.91 (-1.55; -0.27)	0.33	0.0159
**Vaccine Hesitancy group - Doubtful**				
**Randomised condition**				
	2 - 1	0.18 (-0.56; 0.92)	0.38	0.7742
	3 - 1	-0.32 (-1.06; 0.42)	0.38	0.7610
	4 - 1	-0.59 (-1.33; 0.15)	0.38	0.3621
	5 - 1	0.30 (-0.44; 1.05)	0.38	0.7610
	6 - 1	-0.65 (-1.39; 0.09)	0.38	0.3621
	7 - 1	-0.20 (-0.94; 0.54)	0.38	0.7742
	8 - 1	0.15 (-0.58; 0.88)	0.37	0.7742
	9 - 1	-0.62 (-1.36; 0.13)	0.38	0.3621
	10 - 1	-0.05 (-0.80; 0.70)	0.38	0.8901
**Vaccine Hesitancy group - Willing**				
**Randomised condition**				
	2 - 1	0.00 (-0.32; 0.33)	0.16	0.9757
	3 - 1	-0.09 (-0.41; 0.23)	0.16	0.9393
	4 - 1	-0.15 (-0.47; 0.17)	0.16	0.9393
	5 - 1	-0.21 (-0.53; 0.11)	0.16	0.8712
	6 - 1	-0.02 (-0.34; 0.30)	0.16	0.9757
	7 - 1	-0.08 (-0.40; 0.24)	0.16	0.9393
	8 - 1	-0.13 (-0.45; 0.19)	0.16	0.9393
	9 - 1	0.06 (-0.26; 0.38)	0.16	0.9393
	10 - 1	-0.24 (-0.56; 0.08)	0.16	0.8712

* P values adjustment using False Discovery Rate method. C.I. = Confidence intervals. SE = Standard error

4 POST-HOC ANALYSIS

As requested by the reviewer, the primary and secondary outcome analyses were repeated with only participants who have not been vaccinated (n=14,483).

4.1 PRIMARY OUTCOME

4.1.1 Vaccine hesitancy

The primary outcome measure is Oxford Covid-19 Vaccine Hesitancy Measure (Freeman et al, 2020). This is a seven-item scale. Item specific response options (Saris, Revilla, Krosnick & Shaeffer, 2010), coded from 1 to 5, are used. A 'Don't know' option is also provided, which is excluded from scoring. Scores can range between 7 and 35, with higher scores indicating higher COVID-19 vaccine hesitancy.

We checked the assumptions of normality of the model residuals using graphical methods. The outcome residuals were strongly skewed to the right, but the model residuals were found to be sufficiently normally distributed to fit a linear model (Figure S13).

Figure S133. Histogram of residuals from linear regression model

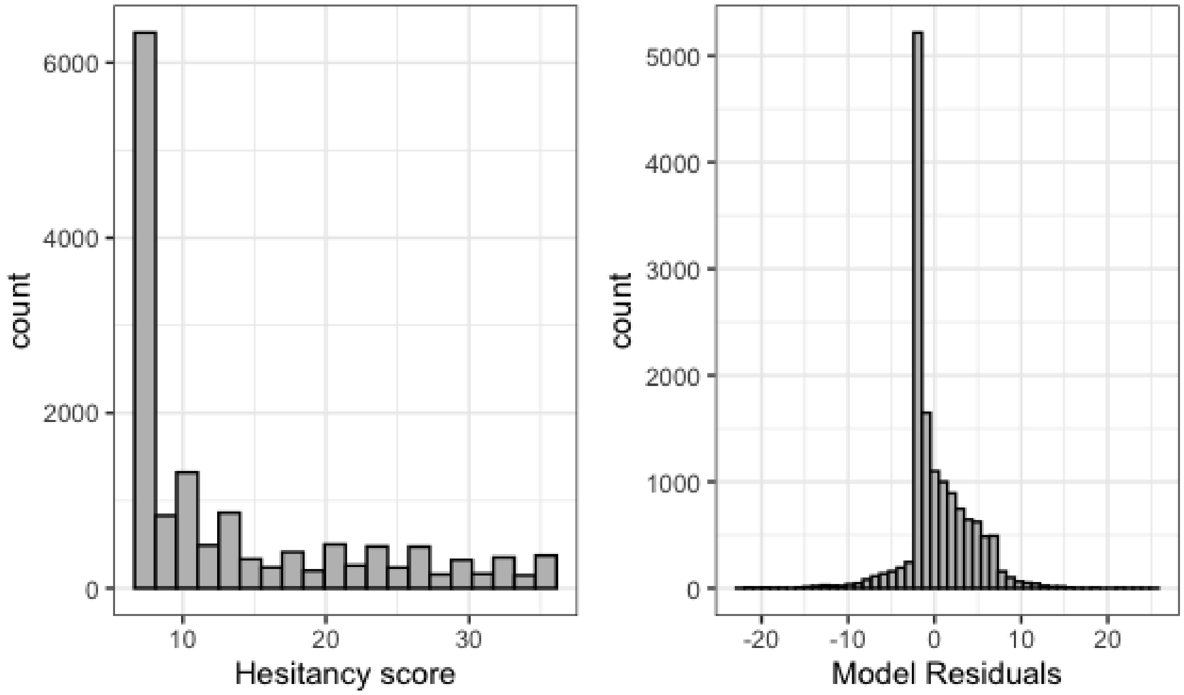

4.1.2 Hesitancy scores between randomised conditions in primary and secondary objectives

The estimated marginal means in each randomised condition and their respective confidence intervals are reported in Table S16.

Table S16. Estimated marginal means in each randomised condition

Randomised Conditions	n	Estimated marginal mean	SE	lower.CI	upper.CI
1	1443	13.79	0.22	13.40	14.20
2	1440	13.77	0.22	13.30	14.20
3	1470	13.84	0.22	13.40	14.30
4	1448	13.63	0.22	13.20	14.10
5	1456	13.58	0.22	13.10	14.00
6	1440	13.71	0.22	13.30	14.10
7	1445	13.71	0.22	13.30	14.10
8	1466	13.60	0.22	13.20	14.00
9	1437	13.97	0.22	13.50	14.40
10	1438	13.41	0.22	13.00	13.80

SE = Standard error

There were no significant differences across the randomised conditions (Table S17).

Table S17. Mean difference between marginal means across randomised condition

Contrast	Estimated mean difference (C.I.)	SE	Adjusted P-value*
2 - 1	-0.02 (-0.63; 0.60)	0.31	0.9548
3 - 1	0.05 (-0.56; 0.66)	0.31	0.9347
4 - 1	-0.16 (-0.78; 0.45)	0.31	0.9093
5 - 1	-0.21 (-0.83; 0.40)	0.31	0.9093
5 - 2	-0.19 (-0.81; 0.42)	0.31	0.9093
5 - 3	-0.26 (-0.87; 0.35)	0.31	0.9093
6 - 1	-0.08 (-0.69; 0.54)	0.31	0.9346
7 - 1	-0.08 (-0.69; 0.54)	0.31	0.9346
8 - 1	-0.19 (-0.80; 0.42)	0.31	0.9093
8 - 7	-0.11 (-0.73; 0.50)	0.31	0.9346
9 - 1	0.18 (-0.44; 0.80)	0.31	0.9093
9 - 4	0.34 (-0.27; 0.96)	0.31	0.9093
9 - 5	0.39 (-0.22; 1.01)	0.31	0.9093
10 - 1	-0.38 (-0.99; 0.24)	0.31	0.9093
10 - 9	-0.56 (-1.18; 0.06)	0.31	0.9093

* P values adjustment using False Discovery Rate method. C.I. = Confidence intervals. SE = Standard error

4.1.3 Impact of hesitancy vaccine levels on reduction of hesitancy scores between Condition 1 (Control) vs Conditions 2, 3, 4, 5, 6, 7, 8

The estimated marginal means in each randomised condition across the vaccine hesitancy groups and their respective confidence intervals are reported in Table S18.

Table S18. Estimated marginal means in each randomised condition across the vaccine hesitancy groups have not been vaccinated

		**n**	**Estimated marginal mean**	**SE**	**lower.CI**	**upper.CI**
**Vaccine Hesitancy group - Strongly hesitant**						
**Randomised condition**						
	1	250	29.05	0.24	28.58	29.53
	2	251	28.44	0.24	27.97	28.91
	3	261	28.28	0.24	27.82	28.74
	4	265	28.07	0.24	27.61	28.53
	5	257	27.30	0.24	26.83	27.76
	6	255	28.39	0.24	27.92	28.86
	7	242	28.26	0.25	27.78	28.75
	8	247	28.05	0.24	27.57	28.53
	9	268	28.46	0.23	28.01	28.92
	10	247	28.09	0.24	27.61	28.57
**Vaccine Hesitancy group - Doubtful**						
**Randomised condition**						
	1	136	20.64	0.33	20.00	21.28
	2	139	20.90	0.32	20.26	21.53
	3	155	20.43	0.31	19.83	21.03
	4	140	20.31	0.32	19.67	20.94
	5	148	20.91	0.31	20.29	21.52
	6	140	20.46	0.32	19.82	21.09
	7	152	20.84	0.31	20.23	21.45
	8	144	21.21	0.32	20.58	21.83
	9	146	19.68	0.32	19.07	20.30
	10	133	20.92	0.33	20.27	21.57
**Vaccine Hesitancy group - Willing**						
**Randomised condition**						
	1	1057	9.30	0.12	9.07	9.53
	2	1050	9.32	0.12	9.09	9.55
	3	1054	9.29	0.12	9.06	9.52
	4	1043	9.06	0.12	8.83	9.29

5	1051	9.19	0.12	8.96	9.42
6	1045	9.23	0.12	9.00	9.46
7	1051	9.33	0.12	9.10	9.56
8	1075	9.26	0.12	9.03	9.49
9	1023	9.36	0.12	9.12	9.59
10	1058	9.04	0.12	8.81	9.27

SE = Standard error

There were significant differences between groups (Condition 3 vs 1), (Condition 4 vs 1), (Condition 5 vs 1), (Condition 7 vs 1) and (Condition 8 vs 1) in the strongly hesitancy group. Participants randomised to condition 3 showed significant reduced levels of hesitancy (mean differences (95% C.I.): -0.77 (-1.44; -0.11); adjusted p-value =0.0373) compared to condition 1. Participants randomised to condition 4 showed significant reduced levels of hesitancy (mean differences (95% C.I.): -0.98 (-1.64; -0.32); adjusted p-value =0.0088) compared to condition 1. Participants randomised to condition 5 showed significant reduced levels of hesitancy (mean differences (95% C.I.): -1.76 (-2.42; -1.09); adjusted p-value = 0.0002) compared to condition 1. Participants randomised to condition 7 showed significant reduced levels of hesitancy (mean differences (95% C.I.): -0.79 (-1.46; -0.11); adjusted p-value =0. 0373) compared to condition 1. Participants randomised to condition 8 showed significant reduced levels of hesitancy (mean differences (95% C.I.): -1.00 (-1.68; -0.33); adjusted p-value =0. 0088) compared to condition 1 (Table S19).

There were no other significant differences in the strongly hesitant group and in the other vaccine hesitancy groups across the randomised conditions [Condition 1 (control) vs Conditions 2, 3, 4, 5, 6, 7, 8].

Table S19. Mean difference between marginal means across randomised conditions

	Contrast	Estimated mean difference (C.I.)	SE	Adjusted P-value*
**Vaccine Hesitancy group - Strongly hesitant**				
**Randomised condition**				
	2 - 1	-0.61 (-1.28; 0.06)	0.34	0.0993
	3 - 1	-0.77 (-1.44; -0.11)	0.34	0.0373
	4 - 1	-0.98 (-1.64; -0.32)	0.34	0.0088
	5 - 1	-1.76 (-2.42; -1.09)	0.34	0.0002
	6 - 1	-0.66 (-1.33; 0.01)	0.34	0.0786
	7 - 1	-0.79 (-1.46; -0.11)	0.35	0.0373
	8 - 1	-1.00 (-1.68; -0.33)	0.34	0.0088
**Vaccine Hesitancy group - Doubtful**				
**Randomised condition**				
	2 - 1	0.26 (-0.64; 1.16)	0.46	0.7409
	3 - 1	-0.21 (-1.09; 0.67)	0.45	0.7409
	4 - 1	-0.33 (-1.23; 0.57)	0.46	0.7409
	5 - 1	0.27 (-0.62; 1.16)	0.45	0.7409
	6 - 1	-0.18 (-1.08; 0.72)	0.46	0.7409

	7 - 1	0.20 (-0.68; 1.09)	0.45	0.7409
	8 - 1	0.57 (-0.33; 1.46)	0.46	0.6339
**Vaccine Hesitancy group - Willing**				
**Randomised condition**				
	2 - 1	0.02 (-0.30; 0.35)	0.17	0.9541
	3 - 1	0.00 (-0.33; 0.32)	0.17	0.9813
	4 - 1	-0.24 (-0.56; 0.09)	0.17	0.5906
	5 - 1	-0.11 (-0.43; 0.22)	0.17	0.9541
	6 - 1	-0.07 (-0.40; 0.26)	0.17	0.9541
	7 - 1	0.03 (-0.30; 0.36)	0.17	0.9541
	8 - 1	-0.04 (-0.36; 0.29)	0.17	0.9541

* P values adjustment using False Discovery Rate method. C.I. = Confidence intervals. SE = Standard error

4.1.4 Impact of hesitancy vaccine levels on reduction of hesitancy scores between Condition 1 (Control) vs Conditions 9 and 10

There was a significant difference between groups (Condition 10 vs 1) in the strongly hesitancy group. Participants randomised to condition 10 showed significant reduced levels of hesitancy (mean differences (95% C.I.): -0.96 (-1.63; -0.29); adjusted p-value = 0. 0107) compared to condition 1 (Table S20).

There were no other significant differences in the strongly hesitant group and in the other vaccine hesitancy groups across the randomised conditions [Condition 1 (control) vs Conditions 9 and 10].

Table S20. Mean difference between marginal means across randomised conditions

	Contrast	Estimated mean difference (C.I.)	SE	Adjusted P-value*
**Vaccine Hesitancy group - Strongly hesitant**				
**Randomised condition**				
	9 - 1	-0.59 (-1.25; 0.07)	0.34	0.0993
	10 - 1	-0.96 (-1.63; -0.29)	0.34	0.0107
**Vaccine Hesitancy group - Doubtful**				
**Randomised condition**				
	9 - 1	-0.95 (-1.85; -0.06)	0.46	0.1800
	10 - 1	0.28 (-0.64; 1.19)	0.47	0.7409
**Vaccine Hesitancy group - Willing**				
**Randomised condition**				
	9 - 1	0.06 (-0.27; 0.39)	0.17	0.9541
	10 - 1	-0.26 (-0.58; 0.07)	0.17	0.5906

* P values adjustment using False Discovery Rate method. C.I. = Confidence intervals. SE = Standard error

4.2 SECONDARY OUTCOME

4.2.1 Impact of hesitancy vaccine levels on reduction of hesitancy scores between Condition 2 vs Conditions 3 and 5

There were significant differences between groups (Condition 5 vs 2) and (Condition 5 vs 3) in the strongly hesitancy group. Participants randomised to condition 5 showed significant reduced levels of hesitancy (mean differences (95% C.I.): -1.15 (-1.81; -0.48); adjusted p-value = 0.0035) compared to condition 2. Participants randomised to condition 5 showed significant reduced levels of hesitancy (mean differences (95% C.I.): -0.98 (-1.64; -0.33); adjusted p-value = 0. 0088) compared to condition 3 (Table S21).

There were no other significant differences in the other vaccine hesitancy groups across the randomised conditions [Condition 5 vs Conditions 2 and 3].

Table S21. Mean difference between marginal means across randomised conditions

Contrast	Estimated mean difference (C.I.)	SE	Adjusted P-value*
**Vaccine Hesitancy group - Strongly hesitant**			
**Randomised condition**			
5 - 2	-1.15 (-1.81; -0.48)	0.34	0.0035
5 - 3	-0.98 (-1.64; -0.33)	0.34	0.0088
**Vaccine Hesitancy group - Doubtful**			
**Randomised condition**			
5 - 2	0.01 (-0.88; 0.89)	0.45	0.9892
5 - 3	0.47 (-0.39; 1.33)	0.44	0.7033
**Vaccine Hesitancy group - Willing**			
**Randomised condition**			
5 - 2	-0.13 (-0.46; 0.20)	0.17	0.9541
5 - 3	-0.10 (-0.43; 0.22)	0.17	0.9541

* P values adjustment using False Discovery Rate method. C.I. = Confidence intervals. SE = Standard error

4.2.2 Impact of hesitancy vaccine levels on reduction of hesitancy scores between Condition 7 and Conditions 8

There were no significant differences in the vaccine hesitancy groups across the randomised conditions [Condition 7 vs Condition 8] (Table S22).

Table S22. Mean difference between marginal means across randomised conditions

Contrast	Estimated mean difference (C.I.)	SE	Adjusted P-value*
**Vaccine Hesitancy group - Strongly hesitant**			
**Randomised condition**			
8 - 7	-0.22 (-0.89; 0.46)	0.35	0.5322
**Vaccine Hesitancy group - Doubtful**			
**Randomised condition**			
8 - 7	0.37 (-0.50; 1.24)	0.44	0.7409
**Vaccine Hesitancy group - Willing**			
**Randomised condition**			
8 - 7	-0.07 (-0.39; 0.26)	0.17	0.9541

* P values adjustment using False Discovery Rate method. C.I. = Confidence intervals. SE = Standard error

4.2.3 Impact of hesitancy vaccine levels on reduction of hesitancy scores between Condition 9 vs Conditions 4 and 5

There was a significant difference between groups (Condition 9 vs 5) in the strongly hesitancy group. Participants randomised to condition 9 showed significant increased levels of hesitancy (mean differences (95% C.I.): 1.17 (0.51; 1.82); adjusted p-value = 0.0035) compared to condition 5 (Table S23).

There were no other significant differences in the strongly hesitant group and in the other vaccine hesitancy groups across the randomised conditions [Condition 9 vs conditions 4 and 5].

Table S23. Mean difference between marginal means across randomised conditions

Contrast	Estimated mean difference (C.I.)	SE	Adjusted P-value*
**Vaccine Hesitancy group – Strongly hesitant**			
**Randomised condition**			
9 – 4	0.39 (-0.25; 1.04)	0.33	0.2690
9 – 5	1.17 (0.51; 1.82)	0.33	0.0035
**Vaccine Hesitancy group – Doubtful**			
**Randomised condition**			
9 – 4	-0.62 (-1.51; 0.26)	0.45	0.6323
9 – 5	-1.22 (-2.09; -0.35)	0.45	0.0533
**Vaccine Hesitancy group – Willing**			
**Randomised condition**			
9 – 4	0.30 (-0.03; 0.62)	0.17	0.5906
9 – 5	0.17 (-0.16; 0.50)	0.17	0.9541

* P values adjustment using False Discovery Rate method. C.I. = Confidence intervals. SE = Standard error

4.2.4 Impact of hesitancy vaccine levels on reduction of hesitancy scores between Condition 9 and Condition 10

There were no significant differences in the vaccine hesitancy groups across the randomised conditions [Condition 10 vs condition 9] (Table S24).

Table S24. Mean difference between marginal means across randomised conditions

	Contrast	Estimated mean difference (C.I.)	SE	Adjusted P-value*
**Vaccine Hesitancy group - Strongly hesitant**				
**Randomised condition**				
	10 - 9	-0.37 (-1.03; 0.29)	0.34	0.2867
**Vaccine Hesitancy group - Doubtful**				
**Randomised condition**				
	10 - 9	1.23 (0.33; 2.13)	0.46	0.0533
**Vaccine Hesitancy group - Willing**				
**Randomised condition**				
	10 - 9	-0.32 (-0.65; 0.01)	0.17	0.5906

* P values adjustment using False Discovery Rate method. C.I. = Confidence intervals. SE = Standard error

5. REFERENCES

Baron, R. M., & Kenny, D. A. (1986). The moderator–mediator variable distinction in social psychological research: Conceptual, strategic, and statistical considerations. *Journal of personality and social psychology*, *51*(6), 1173.

Barrett, P. (2007). Structural equation modelling: Adjudging model fit. Personality And Individual Differences, 42(5), 815-824

Buuren, S. V., & Groothuis-Oudshoorn, K. (2010). mice: Multivariate imputation by chained equations in R. *Journal of statistical software*, 1-68.

Byrne, B. (1989). A primer of LISREL. New York: Springer

Freeman, D., Loe, B. S., Chadwick, A., Vaccari, C., Waite, F., Rosebrock, L., ... & Lambe, S. (2020). COVID-19 vaccine hesitancy in the UK: the Oxford coronavirus explanations, attitudes, and narratives survey (Oceans) II. *Psychological medicine*, 1-15

Hayes, A. F., & Preacher, K. J. (2014). Statistical mediation analysis with a multicategorical independent variable. *British journal of mathematical and statistical psychology*, *67*(3), 451-470.

Jorgensen, T. D., Pornprasertmanit, S., Schoemann, A. M., Rosseel, Y., Miller, P., Quick, C., ... & Enders, C. (2016). Package 'semTools'.

Lenth, R., Singmann, H., Love, J., Buerkner, P., & Herve, M. (2018). Emmeans: Estimated marginal means, aka least-squares means. *R package version*, *1*(1), 3.

MacKinnon, D. P., Lockwood, C. M., & Williams, J. (2004). Confidence limits for the indirect effect: Distribution of the product and resampling methods. *Multivariate behavioral research*, *39*(1), 99-128.

Marsh, H., & Hocevar, D. (1985). Application of confirmatory factor analysis to the study of self-concept: First- and higher order factor models and their invariance across groups. *Psychological Bulletin*, *97*(3), 562-582.

Preacher, K. J., & Selig, J. P. (2012). Advantages of Monte Carlo confidence intervals for indirect effects. *Communication Methods and Measures*, *6*(2), 77-98.

R Core Team (2020). R: A language and environment for statistical computing. R Foundation for Statistical Computing, Vienna, Austria. URL https://www.R-project.org/.

Rosseel, Y. (2012). Lavaan: An R package for structural equation modeling and more. Version 0.5–12 (BETA). *Journal of statistical software*, *48*(2), 1-36.

Rubin, D.B. (1987). Multiple Imputation for Nonresponse in Surveys. J. Wiley & Sons, New York.

Schafer, J. L. (1999). Multiple imputation: a primer. *Statistical methods in medical research*, *8*(1), 3-15.

Wheaton, B., Muthen, B., Alwin, D. F., & Summers, G. F. (1977). Assessing reliability and stability in panel models. Sociological methodology, 8, 84-136.

6. APPENDICES

Appendix I. CONSORT 2010 Flow Diagram

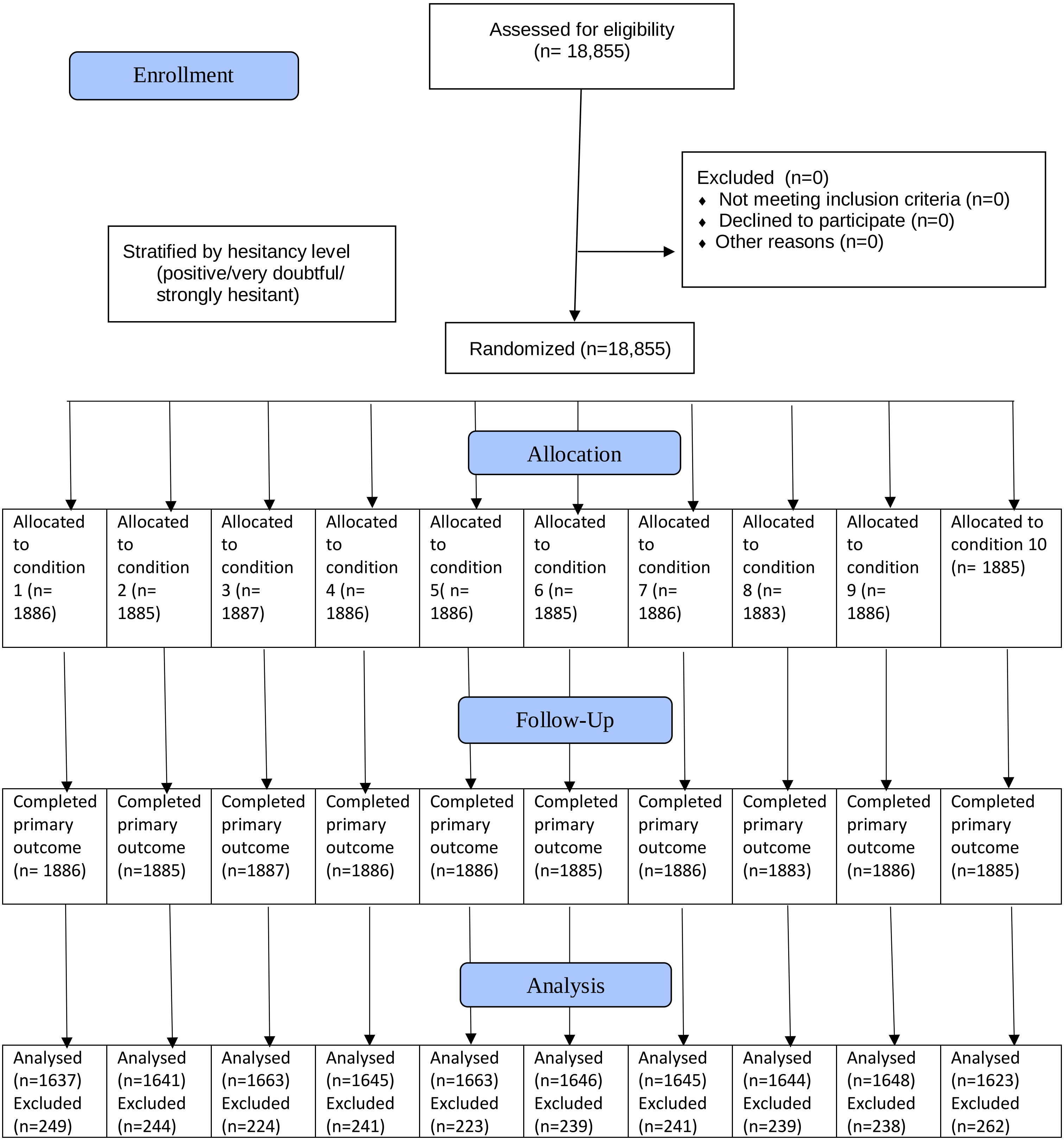

Appendix II. Data cleaning done by the crowd sourcing platform (LUCID)

Data Cleaning

Once data collection is underway, Lucid can provide data cleaning or scrubbing, which is the act of removing responses for gibberish, illogical, or fraudulent behavior. Data cleaning is conducted in real-time, while the survey is being fielded to ensure the final dataset is clean and ready for analysis once data collection is complete.

When Lucid is handling programming, the data should always be cleaned. Lucid PSMs can clean out obvious bad verbatims and speeders based on median LOI (removal of any cases that have completed the survey in less than ⅓ of the median LOI).

Below are the types of respondents removed:

1. Bad Verbatims: Respondents who type in gibberish, vulgar, or illogical responses to open ended questions
2. Straightliners/Flatliners: Respondents who answer grid questions in the same manner (i.e. all select "3") or create patterns in grids
3. Speeders: Respondents who speed through the survey. Completion time of ⅓ of the median LOI or below is the standard best practice.
4. Duplicates: Identification of respondents who have taken the survey multiple times - IP Address, Cookie ID, etc.
5. Bots: Software created with the intention of gaming surveys for incentives. Identified by finding verbatim, demographic, attitudinal patterns, and geo-location data.

Table S25. Beliefs about COVID-19 vaccination (Oxford Vaccine Confidence and Complacency Scale) by hesitancy stratification level.

Belief	Group	n	Mean	SD	df	F	p-value
Collective Benefit*	Willing	11156	8.23	2.29	2,14658	6792.63	<.001
	Doubtful	1574	12.33	3.36			
	Strongly hesitant	1931	16.02	4.82			
Risk and efficacy*	Willing	9670	6.64	1.61	2, 14102	4948.93	<.001
	Doubtful	1980	8.83	1.83			
	Strongly hesitant	2455	10.54	2.53			
Speed of development*	Willing	11124	6.14	2.06	2,15404	4933.47	<.001
	Doubtful	1755	8.63	2.23			
	Strongly hesitant	2528	10.73	2.70			
Side effects*	Willing	10883	5.25	1.72	2,15077	6185.20	<.001
	Doubtful	1842	8.05	2.24			
	Strongly hesitant	2355	9.92	2.75			

* All group scores differ at p<.001.

Information conditions.

1. Control (sentences from NHS information provision)

The coronavirus (COVID-19) vaccines are safe and effective, and give you the best protection against coronavirus. They have been approved by the independent Medicines and Healthcare products Regulatory Agency (MHRA).

2. Collective benefit (a) (concerning benefit of not getting ill)

As we all know, coronavirus has had a dramatic effect on the UK, putting severe strain on families, health services, schools and universities, and businesses. Virtually every sector of our society and economy has been affected, bringing disruption and inconvenience to us all. Every case of COVID-19, whether it results in hospitalisation, long-term health issues, or simply a period of isolation, causes more problems. We're all eager to get back to the way things were before the pandemic struck. By making sure we're vaccinated -- and therefore less likely to become ill with COVID-19 -- we can play our part in helping the country to bounce back as quickly as possible.

The coronavirus (COVID-19) vaccines are safe and effective, and give you the best protection against coronavirus. They have been approved by the independent Medicines and Healthcare products Regulatory Agency (MHRA).

3. Collective benefit (b) (concerning benefit of not spreading virus)

The vaccines make it less likely that we'll pass on COVID-19 to other people.
This means that by being vaccinated we're helping to protect each other: family, friends, neighbours, and colleagues. It's all the more important given that some of the people we meet may be especially vulnerable to the effects of the virus -- though we might not be able to tell just by looking at them.

The sooner we're all vaccinated, the sooner life will return to normal. The economy will be properly up and running again; children and young people will be able to attend school and university; and we can all get back to the activities we used to enjoy, like getting together with family and friends, going to the cinema or pub, or taking a holiday overseas. In fact, if we're all vaccinated cases of COVID-19 are expected to decline substantially, just like diseases such as smallpox, polio, and tetanus that used to kill or disable millions of people.

The coronavirus (COVID-19) vaccines are safe and effective, and give you the best protection against coronavirus. They have been approved by the independent Medicines and Healthcare products Regulatory Agency (MHRA).

4. Full collective benefits (combining a) and b))

The vaccines make it less likely that we'll pass on COVID-19 to other people.
This means that by being vaccinated we're helping to protect each other: family, friends, neighbours, and colleagues. It's all the more important given that some of the people we meet may be especially vulnerable to the effects of the virus -- though we might not be able to tell just by looking at them.

The sooner we're all vaccinated, the sooner life will return to normal. The economy will be properly up and running again; children and young people will be able to attend school and university; and we can all get back to the activities we used to enjoy, like getting together with family and friends, going to the cinema or pub, or taking a holiday overseas. In fact, if we're all vaccinated cases of COVID-19 are expected to decline substantially, just like diseases such as smallpox, polio, and tetanus that used to kill or disable millions of people.

As we all know, coronavirus has had a dramatic effect on the UK, putting severe strain on families, health services, schools and universities, and businesses. Virtually every sector of our society and economy has been affected, bringing disruption and inconvenience to us all. Every case of COVID-19, whether it results in hospitalisation, long-term health issues, or simply a period of isolation, causes more problems. We're all eager to get back to the way things were before the pandemic struck. By making sure we're vaccinated -- and therefore less likely to become ill with COVID-19 -- we can play our part in helping the country to bounce back as quickly as possible.

The coronavirus (COVID-19) vaccines are safe and effective, and give you the best protection against coronavirus. They have been approved by the independent Medicines and Healthcare products Regulatory Agency (MHRA).

5. Personal benefit

Catching coronavirus can seriously disrupt your life. It can take you away from work, education, family, and friends. Even mild symptoms mean you'll be required to isolate for ten days, with all the inconvenience that brings. And you can't be sure, even if you're relatively young and fit, that you won't be seriously ill or struggle with long-term COVID-related problems: as many as one in five people are still unwell five weeks after contracting COVID-19; one in ten are still experiencing symptoms three months later. Vaccination minimises the chances of you falling ill with COVID-19, so you won't need to worry about what the virus might have in store for you.

The coronavirus (COVID-19) vaccines are safe and effective, and give you the best protection against coronavirus. They have been approved by the independent Medicines and Healthcare products Regulatory Agency (MHRA).

6. Seriousness

We shouldn't underestimate the seriousness of COVID-19. Even after months of lockdown restrictions, Public Health England has reported that by the first week of January 78,508 people in the UK had died from the illness and 297,000 had been hospitalised. Deaths from COVID-19 already far outstrip the annual average of 28,500 we've seen from flu and pneumonia over the last twenty years. The number hospitalised is already more than eight times what we'd expect per year with flu. For many of us, the effects of falling ill with COVID-19 stick around: as many as one in five people are still unwell five weeks after diagnosis; one in ten are still experiencing symptoms three months later.

The coronavirus (COVID-19) vaccines are safe and effective, and give you the best protection against coronavirus. They have been approved by the independent Medicines and Healthcare products Regulatory Agency (MHRA).

7. Safety/speed (addressing speed issues directly)

Some people may worry that the vaccines have been developed too quickly to be safe. The speed of development reflects exceptional commitment, investment, and co-operation from scientists, governments, public health organisations, pharmaceutical companies -- and tens of thousands of members of the public who volunteered to test the vaccines.

Some may be concerned that the vaccines haven't been tested for long enough. Decades of vaccine research show that side effects don't suddenly appear months and years after vaccination. Because of the way vaccines work -- quickly training the body's immune system to fight off a virus -- any issues arise within a month and usually much sooner. No serious problems have been reported by any of the thousands of people who have taken the COVID-19 vaccines so far. Any side effects are typically mild (for example a sore arm or headache), and last less than a week.

The speed of the vaccines' development hasn't affected the approval process: they have undergone the same rigorous, independent assessment as any other medicine.

The coronavirus (COVID-19) vaccines are safe and effective, and give you the best protection against coronavirus. They have been approved by the independent Medicines and Healthcare products Regulatory Agency (MHRA).

8. Safety/rigour (addressing speed issues indirectly)

The COVID-19 vaccines result from exceptional levels of commitment, investment, and co-operation from scientists, governments, public health organisations, pharmaceutical companies -- and tens of thousands of members of the public who volunteered to test them.

Decades of vaccine research have shown that side effects don't suddenly appear months and years after vaccination. Because of the way vaccines work -- quickly training the body's immune system to be ready to fight off a virus -- any issues show up within a month and usually much sooner. No serious problems have been reported by any of the thousands of people who have taken the COVID-19 vaccines so far. Any side effects are typically mild (for example a sore arm or headache), and last less than a week.

The vaccines have had to undergo the same rigorous, independent approval process as any other medicine. Large-scale clinical trials involving tens of thousands of people have shown that the vaccines will protect the vast majority of people from getting COVID-19.

The coronavirus (COVID-19) vaccines are safe and effective, and give you the best protection against coronavirus. They have been approved by the independent Medicines and Healthcare products Regulatory Agency (MHRA).

9. Collective and personal benefits (combining 4 and 5)

The vaccines make it less likely that we'll pass on COVID-19 to other people.
This means that by being vaccinated we're helping to protect each other: family, friends, neighbours, and colleagues. It's all the more important given that some of the people we meet may be especially vulnerable to the effects of the virus -- though we might not be able to tell just by looking at them.

The sooner we're all vaccinated, the sooner life will return to normal. The economy will be properly up and running again; children and young people will be able to attend school and university; and we can all get back to the activities we used to enjoy, like getting together with family and friends, going to the cinema or pub, or taking a holiday overseas. In fact, if we're all vaccinated cases of COVID-19 are expected to decline substantially, just like diseases such as smallpox, polio, and tetanus that used to kill or disable millions of people.

As we all know, coronavirus has had a dramatic effect on the UK, putting severe strain on families, health services, schools and universities, and businesses. Virtually every sector of our society and economy has been affected, bringing disruption and inconvenience to us all. Every case of COVID-19, whether it results in hospitalisation, long-term health issues, or simply a period of isolation, causes more problems. We're all eager to get back to the way things were before the pandemic struck. By making sure we're vaccinated -- and therefore less likely to become ill with COVID-19 -- we can play our part in helping the country to bounce back as quickly as possible.

Catching coronavirus can seriously disrupt your life. It can take you away from work, education, family, and friends. Even mild symptoms mean you'll be required to isolate for ten days, with all the inconvenience that brings. And you can't be sure, even if you're relatively young and fit, that you won't be seriously ill or struggle with long-term COVID-related problems: as many as one in five people are still unwell five weeks after contracting COVID-19; one in ten are still experiencing symptoms three months later. Vaccination minimises the

chances of you falling ill with COVID-19, so you won't need to worry about what the virus might have in store for you.

The coronavirus (COVID-19) vaccines are safe and effective, and give you the best protection against coronavirus. They have been approved by the independent Medicines and Healthcare products Regulatory Agency (MHRA).

10. Full text (combining 6, 8, and 9)

The vaccines make it less likely that we'll pass on COVID-19 to other people.
This means that by being vaccinated we're helping to protect each other: family, friends, neighbours, and colleagues. It's all the more important given that some of the people we meet may be especially vulnerable to the effects of the virus -- though we might not be able to tell just by looking at them.

The sooner we're all vaccinated, the sooner life will return to normal. The economy will be properly up and running again; children and young people will be able to attend school and university; and we can all get back to the activities we used to enjoy, like getting together with family and friends, going to the cinema or pub, or taking a holiday overseas. In fact, if we're all vaccinated cases of COVID-19 are expected to decline substantially, just like diseases such as smallpox, polio, and tetanus that used to kill or disable millions of people.

As we all know, coronavirus has had a dramatic effect on the UK, putting severe strain on families, health services, schools and universities, and businesses. Virtually every sector of our society and economy has been affected, bringing disruption and inconvenience to us all. Every case of COVID-19, whether it results in hospitalisation, long-term health issues, or simply a period of isolation, causes more problems. We're all eager to get back to the way things were before the pandemic struck. By making sure we're vaccinated -- and therefore less likely to become ill with COVID-19 -- we can play our part in helping the country to bounce back as quickly as possible.

Catching coronavirus can seriously disrupt your life. It can take you away from work, education, family, and friends. Even mild symptoms mean you'll be required to isolate for ten days, with all the inconvenience that brings. And you can't be sure, even if you're relatively young and fit, that you won't be seriously ill or struggle with long-term COVID-related problems: as many as one in five people are still unwell five weeks after contracting COVID-19; one in ten are still experiencing symptoms three months later. Vaccination minimises the chances of you falling ill with COVID-19, so you won't need to worry about what the virus might have in store for you.

We shouldn't underestimate the seriousness of COVID-19. Even after months of lockdown restrictions, Public Health England has reported that by the first week of January 78,508 people in the UK had died from the illness and 297,000 had been hospitalised. Deaths from COVID-19 already far outstrip the annual average of 28,500 we've seen from flu and pneumonia over the last twenty years. The number hospitalised is already more than eight times what we'd expect per year with flu.

The COVID-19 vaccines result from exceptional levels of commitment, investment, and co-operation from scientists, governments, public health organisations, pharmaceutical companies -- and tens of thousands of members of the public who volunteered to test them.

Decades of vaccine research have shown that side effects don't suddenly appear months and years after vaccination. Because of the way vaccines work -- quickly training the body's immune system to be ready to fight off a virus -- any issues show up within a month and usually much sooner. No serious problems have been reported by any of the thousands of people who have taken the COVID-19 vaccines so far. Any side effects are typically mild (for example a sore arm or headache), and last less than a week.

The vaccines have had to undergo the same rigorous, independent approval process as any other medicine. Large-scale clinical trials involving tens of thousands of people have shown that the vaccines will protect the vast majority of people from getting COVID-19.

The coronavirus (COVID-19) vaccines are safe and effective, and give you the best protection against coronavirus. They have been approved by the independent Medicines and Healthcare products Regulatory Agency (MHRA).
